# An Analytical Understanding of Reentrant Condensation of a Polyelectrolyte in the Presence of an Oppositely Charged Surfactant

**DOI:** 10.1021/acs.langmuir.5c01217

**Published:** 2025-07-22

**Authors:** Huaisong Yong, Holger Merlitz

**Affiliations:** † School of New Energy and Materials, 12466 Southwest Petroleum University, Chengdu 610500, China; ‡ Department of Molecules & Materials, MESA+ Institute, 3230 University of Twente, Enschede 7500 AE, The Netherlands; § Institute Theory of Polymers, Leibniz-Institut für Polymerforschung Dresden e.V., Dresden D-01069, Germany

## Abstract

We explore the phase-transition mechanism of the reentrant condensation of a polyelectrolyte in the presence of an oppositely charged surfactant, which is of fundamental importance to the understanding of liquid–liquid phase separation (LLPS) in soft materials and biological systems. We focus on the adsorption and attraction effects of surfactants near/on polymer chains and ignore their own nonessential mixing effects if surfactant molecules are far away from polymer chains. This novel approach allows us to construct a simple mean-field equilibrium theory with closed-form analytical solutions, which can rationalize the essential features of the emergent “egg shape”-like phase diagram. The theory addresses that a strong electrostatic adsorption between the ionic monomers and surfactant ions is critical to understand the peculiar phenomenon that both the collapse and re-entry transitions of polyelectrolytes can occur when the concentration of the surfactant is lower than its bulk critical micelle concentration (CMC). Our theory also indicates that a minimum coupling energy for the nonlinear hydrophobic-aggregation effect of the adsorbed surfactant is essential for a phase transition to occur, which explains why polyelectrolytes show such a phase transition only if the surfactant chain length is beyond a minimum value. This work provides insight into the understanding of liquid–liquid phase separation in biological systems if proteins and/or peptides bound to DNAs and/or RNAs play an important role.

## Graphical abstract

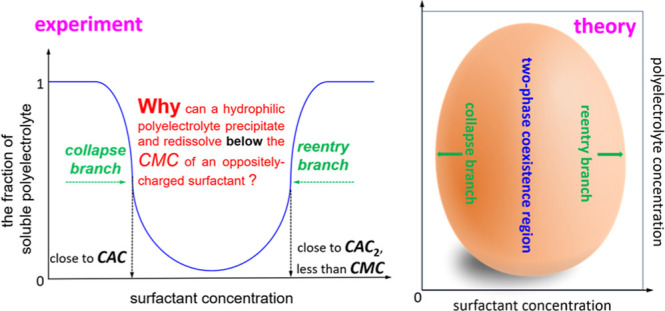

## 1 Introduction

Phase transitions of polyelectrolyte solutions are pivotal to the stability and function of soft materials. The predictability of phase separations such as condensation and aggregation in polyelectrolyte solutions is of fundamental importance to various fields, from materials science^1–3^ to cell biology^4,5^ and art preservation.^6,7^ A particular scenario of polyelectrolyte phase separations is the reentrant condensation of a polyelectrolyte in the diluted aqueous solution of an oppositely charged surfactant, as sketched in Figure 1a, which has attracted significant attention in the past decades.^8–16^ A remarkable feature of the reentrant condensation is that both the collapse and the reentry branches occur at rather low surfactant concentrations,^17–20^ where the overall concentration of the added surfactant (such as hexadecyl trimethylammonium bromides) is usually on the order of 5 mmol/L and thus below the bulk critical micelle concentration (CMC) of the surfactant. It is worth mentioning that this reentrant condensation cannot be well-understood merely according to classical polyelectrolyte theories,^21–23^ which predict the absence of any reentrant signature for polyelectrolyte phase transitions when the concentration of added small monovalent salts is on the order of 5 mmol/L. Therefore, new concepts and theoretical formalisms are required to understand this observed peculiar reentrant behavior at low-surfactant concentrations.

**Figure 1:**
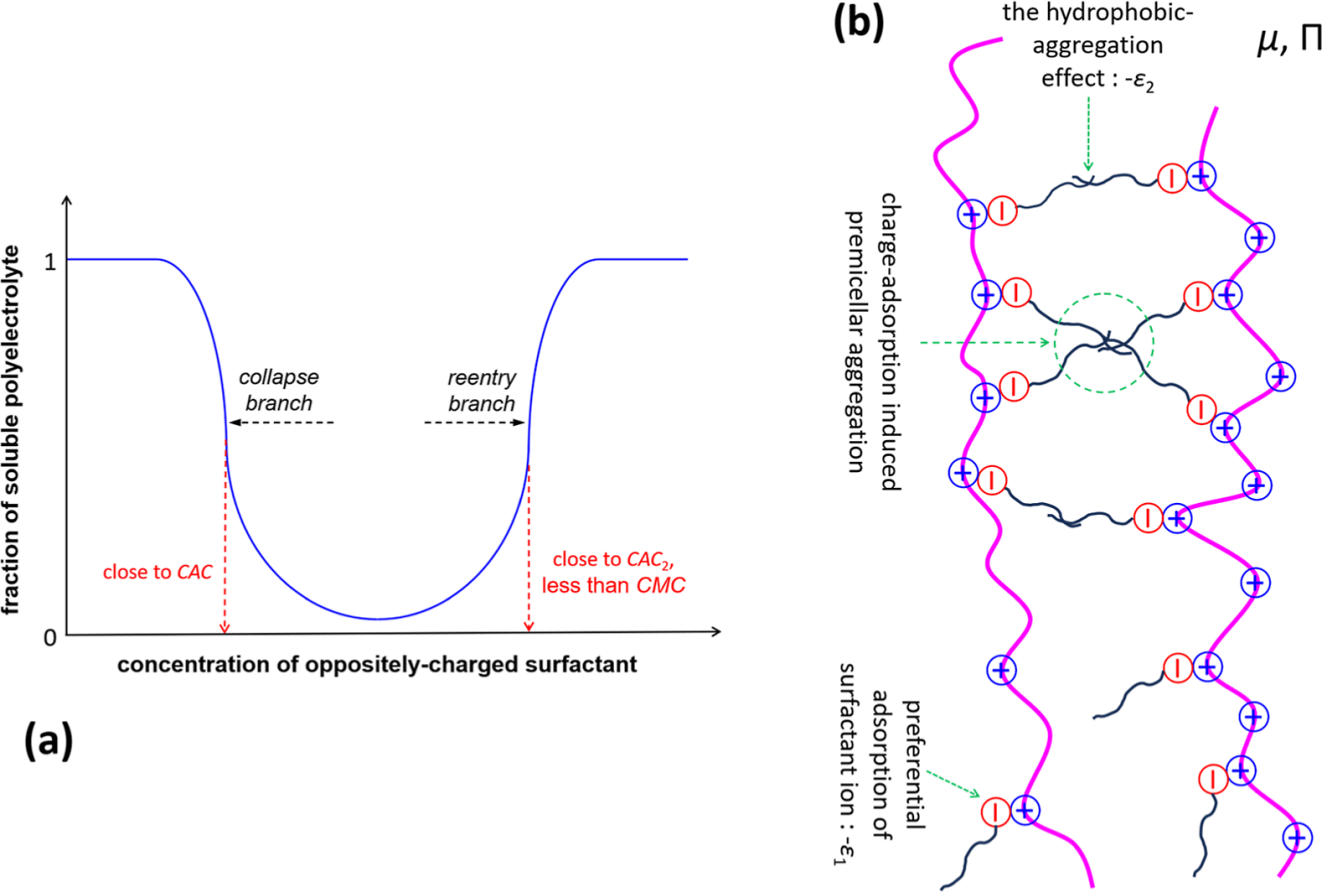
(a) A sketch of the reentrant condensation of polyelectrolytes induced by a diluted oppositely charged surfactant. Typical values of surfactant concentrations for collapse and reentry transitions are on the order of 1 mmol/L and below the CMC of the surfactant.^17–20^ Note that the reentrant condensation is not necessarily symmetric with respect to surfactant concentrations. (b) A sketch of the well-studied phase-transition mechanism of electrostatic-adsorption-induced hydrophobic aggregation^20,25–28^ for the collapse branch of the reentrant condensation as illustrated in panel (a). The ionic monomers (symbol “⊕” in the figure) are preferentially adsorbed by oppositely charged surfactant ions (symbol “⊖” in the figure), and polyelectrolyte chains further form temporary bridges due to the hydrophobic-aggregation effect of adsorbed surfactant ions (black lines which connect in the figure). The pink lines represent polymer chains, and we do not show counterions of both polyelectrolytes and surfactants for simplification.

Another unusual feature of the reentrant condensation is that the electrostatic binding between the charged monomer and the oppositely charged surfactant leads to the formation of insoluble polymer–surfactant coacervates once a certain surfactant concentration, known as the critical aggregation concentration (CAC), is exceeded. The CAC can be several orders of magnitude below the CMC of the surfactant,^24^ which implies that the influence of the bulk micellar behaviors of the surfactant is negligible for the reentrant condensation. We note that in the literature,^19,20^ a surfactant concentration at which the insoluble polymer–surfactant coacervates start to redissolve (i.e., the onset of the reentry branch of the polyelectrolyte condensation in Figure 1a) is usually termed the second critical aggregation concentration (CAC_2_).

Clarifying the function of oppositely charged surfactants in such a reentrant condensation (as sketched in Figure 1a) is critical for a deep understanding of biological phase separations if proteins/peptides bound to DNAs/RNAs play an important role,^29–37^ for example, for a better understanding of the DNA length on the co-condensation behaviors of proteins with DNAs.^29,35^ Furthermore, from the aspect of applied research, the elucidation of the phase-transition mechanism of the reentrant condensation is pivotal for developing new polymer formulations such as for drug delivery^10^ and new soft materials such as porous polymer materials.^38^

A well-studied phase-transition mechanism^20,25–28^ for the collapse branch of the reentrant condensation (as sketched in Figure 1a) is that the surfactant ion replaces the polyelectrolyte counterions and preferentially adsorbs on the ionic monomers as well as forming electrostatic dipoles. Furthermore, the long hydrophobic tails of the surfactants can undergo hydrophobic aggregation (Figure 1b). Thus, when the surfactant concentration reaches a critical value, a charge-adsorption-induced premicellar aggregation occurs, which ultimately leads to the formation of insoluble polymer–surfactant coacervates. In contrast, the phase-transition mechanism for the reentry branch, as sketched in Figure 1a, can be significantly complicated by specific chemistry-related details of the uncharged moieties of the polyelectrolyte. It has been reported that the hydrophobicity of uncharged monomers of the polyelectrolyte affects and decreases the surfactant concentration that is necessary to trigger the reentry transition.^20,39^

Related to the nonlinear attraction effect among ionic monomers deriving from the electrostatic adsorption of the surfactant^20,25–28^ (as illustrated by Figure 1b), theoretical rationalizations in the past decades such as chemical reaction-like theories^40,41^ and lattice/field-like theories^18,28,40,42–53^ have significantly promoted the understanding of phase behaviors of polyelectrolytes in the presence of oppositely charged surfactants, such as recent interesting studies on the complicated micellar structures^41–44^ and the nonequilibrium/metastable/kinetically trapped states^8,9,11^ of polymer–surfactant coacervates. However, there remain two fundamental problems^54^ to be addressed clearly for the reentrant condensation as sketched in Figure 1a: the first problem is why polyelectrolytes show phase transition only if the surfactant chain length is longer than a minimum length.^55–58^ The second problem is why both the collapse and re-entry transitions of polyelectrolytes can occur when the concentration of the surfactant is lower than its bulk critical micelle concentration (CMC).

The primary goal of this work is to try to resolve the above two problems by exploiting the well-studied phase-transition mechanism, as sketched in Figure 1b. Compared with other published works,^18,28,40–53^ the novelty of the present work lies in the selection of the contributions to the free energy function. Experiments have shown that a reentrant condensation of a polyelectrolyte can take place when the surfactant concentration is several orders of magnitude below its bulk critical micelle concentration (CMC).^24^ Thus, although the surfactant’s own entropic mixing term is included in the construction of the free energy function in our mean-field equilibrium theory, it would significantly increase the complexity of the subsequent analytical description without actually contributing to the reentrant phenomenon that we want to describe. We therefore omitted this term in the subsequent analytical calculation. Instead, we focus on an explicit contribution from the hydrophobic interaction among the tails of the adsorbed surfactants, which turns out to play a pivotal role in the properties of the resulting phase transition. This novel approach allows us to successfully solve our theory analytically with closed-form solutions and to rationalize the essential features such as the emergent “egg shape” of spinodal and binodal phase diagrams for the reentrant condensation, as illustrated by Figure 1a.

In the rest of this article, a mean-field theory for the polyelectrolyte solution in terms of the free energy is constructed in Section 2. Its analytical solution will be considered in detail in Section 3, where we outline some general results of mean-field theory. Furthermore, a simplified phase diagram of the polyelectrolyte solution will be discussed in this section, as well as the applicability of the mean-field theory. Final concluding remarks are given in Section 4.

## 2 Methods and Theory

In this section, we construct the mean-field theory as shown by eq 1 for the polyelectrolyte solution in the presence of an oppositely charged surfactant based upon the Gibbs free energy and clarify its physics foundation. The theory is unfolded as follows.

As sketched in Figure 1b, we consider flexible polyelectrolytes with monovalent ionic monomers and monovalent counterions in an aqueous solution. We denote *N* as the degree of polymerization of the polyelectrolyte chain and *a* as the extent of each monomer along the direction of the polymer backbone. The charged monomers are distributed randomly on the polyelectrolyte chain, and their fraction is denoted by *p*. In general, the size of an ionized monomer, the size of a counterion of polyelectrolyte, the size of a counterion of the surfactant, and the size of a solvent molecule can be very different. Since we are interested in the general physical understanding of the theory, we restrict ourselves here to the symmetric case, i.e., we consider that these sizes are identical in the spirit of the classical Flory–Huggins lattice model,^59,60^ which simplifies the analytical arguments substantially. The overall volume fraction of monovalent ionic monomers and neutral monomers is denoted by *c*, so that the volume fraction of monovalent counterions is *pc*. We denote the volume fraction of the counterions of the surfactant as *c*_*x*_, and then the volume fraction of the surfactant ion is *nc*_*x*_, where *n* denotes the surfactant chain length in units of a polyelectrolyte monomer.

Here and in the following, let us consider the free energy per unit of volume for an incompressible system if not otherwise noted. The volume unit is given by the size of the solvent molecules in the spirit of the classical Flory–Huggins lattice model.^59,60^ We separate the system into two parts: the polymer chains with their enclosed solvents and small ions and the bulk without polymer chains. The contribution to the free energy from the three-dimensional mixing of polymers with the solvent and the added surfactant is given by *G*_sol_, see the first line of eq 1. The bulk is just described by an osmotic pressure (∏) acting on the polymer phase by the bulk. If the volume of the polymer coils changes, then mechanical work against the external pressures is involved in the free energy change. This will become important when we minimize the free energy with respect to the monomer concentration (*c*). This approach is thus based on the framework of the isothermal–isobaric (*NPT*) ensemble. Here and in the following, we consider energies in units of *k*_B_ *T*, where *k*_B_ is the Boltzmann constant and *T* is the thermodynamic temperature.

1 $$\begin{array}{l} G_{sol}=\frac{c}{N}\ln\left(c\right)+pc\;\ln\left(pc\right)+c_{x}\;\ln\left(c_{x}\right)+c_{x}\;\ln\left(nc_{x}\right)\\ +\left[1-\left(1+p\right)c-\left(1+n\right)c_{x}\right]\ln\left[1-\left(1+p\right)c-\left(1+n\right)c_{x}\right]+\Pi \\ G_{FH}=\left[1-\left(1+p\right)c-\left(1+n\right)c_{x}\right]\left[p\varepsilon_{FH,1}+\left(1-p\right)\varepsilon_{FH,2}\right]c\\ \frac{G_{ads}}{pc}=\frac{\varphi }{\lambda }\ln\left(\varphi \right)+\left(1-\varphi \right)\ln\left(1-\varphi \right)-\mu \varphi -\varepsilon_{1}\frac{\varphi }{\lambda }+\chi_{s}\varphi \left(1-\varphi \right)\\ \frac{G_{attr}}{pc}=-\varepsilon_{2}\varphi^{2}\left(1-\rho \right)\left(pc\right)\approx -\varepsilon_{2}\varphi^{2}\left(1-\varphi \right)\left(pc\right)\\ G_{DS}≃-\frac{{\left(\kappa a\right)}^{3}}{8\pi \left(1+\kappa a\right)}\approx -\frac{{\left(\kappa a\right)}^{3}}{8\pi }=-\sqrt{\pi }{\left(\frac{l_{B}}{a}\right)}^{3/2}{\left[pc+\left(1+n\right)c_{x}\right]}^{3/2}\\ G\left(\varphi ,c,c_{x}\right)=G_{sol}+G_{FH}+G_{ads}+G_{attr}+G_{DS} \end{array}$$

The energy of nonelectrostatic excluded-volume interactions between the solvent (water) and the charge-neutral part of monomers is given by the classical Flory–Huggins formalism^59,60^ and denoted as *G*_FH_, see the second line of eq 1. The Flory–Huggins parameters between the solvent and charged monomers are denoted by ε_FH,1_, and ε_FH,2_ are the Flory–Huggins parameters between the solvent and uncharged monomers. In experiments, these Flory–Huggins parameters can be directly estimated by using Hansen solubility parameters^61^ according to molecular structures of both monomers and solvents.

The isothermal adsorption free energy per unit of volume owing to the one-dimensional mixing of surfactant ions and monovalent polyelectrolyte counterions on ionic monomers is given by *G*_ads_, see the third line of eq 1. The fraction of ionic monomers occupied preferentially by the large surfactant ions is denoted by φ. The excess-adsorption strength of one surfactant ion compared to a monovalent counterion of the polyelectrolyte on its ionic monomers is denoted by ε_1_. The exchange chemical potential of a surfactant ion on the polymer chains is denoted by μ, which scales as μ ∼ ln­(*nc*_*x*_) if the surfactant concentration (*n* + 1)*c*_*x*_ in the bulk is sufficiently small. Because there is a strong demixing tendency between the surfactant and solvent molecules, we take care of this demixing effect on polyelectrolyte chains by the parameter χ_s_, which can be as large as 2.0 for alkyl trimethylammonium bromides.^62^ We denote the volume ratio between the ionic head of a surfactant and a solvent (or a counterion) by λ, which is usually on the order of unity, though it can be varied significantly in some specific experiments.^63,64^

As sketched in Figure 1b, we consider the associative attraction, *G*_attr_, between ionized monomers caused by temporary bridges due to the hydrophobic-aggregation effect of adsorbed surfactant ions on polymer chains, see the fourth line of eq 1. Here, the bridge is a kind of short-range attractive interaction derived from the formation of electrostatic dipoles between monovalent surfactant ions and monovalent ionic monomers, which leads us to a rather simple statistical construction of *G*_attr_. Notice that unbound surfactant molecules can also assemble hydrophobically with a bound surfactant molecule. Thus, the presence of a free surfactant molecule close to polymer chains will inevitably frustrate the effective temporary cross-linking effect. Under a mean-field consideration, we assume that a bound surfactant molecule meets a second bound surfactant molecule with a probability (φ^2^ *c*). However, the effective bridge can be formed only if free surfactant molecules are not present, which is expressed by the probability (1 – ρ), where ρ is the volume fraction of the surfactant ion close to polyelectrolyte chains in the solvent phase. The coupling energy (ε_2_) stands for the hydrophobic energy gain per surfactant that is adsorbed on a polyelectrolyte chain. Since this hydrophobic energy gain originates in the overlap between hydrophobic tails (see Figure 1b), it increases with the degree of polymerization of the surfactant (*n*). Thus, ε_2_ can be viewed as being a parameter which depends on *n* but is independent of the solution concentration without restricting generality.

It is worth noting that there is a significant enrichment of oppositely charged surfactants in networks/gels in the phase transition of polyelectrolyte networks/gels, which was found by Khokhlov^53^ and Rumyantsev^47^ with their co-workers. We also note experiments^65^ showed that the preferential adsorption of large-sized surfactant ions on ionic monomers inevitably leads to the enrichment of surfactant ions around polyelectrolyte chains, which indicates that there is a relation of *nc*_*x*_ ≪ ρ → φ. To simplify our following analytical calculations, these facts prompt us to assume that the saturation of the bound surfactant on polyelectrolyte chains in semidilute and concentrated polymer solutions is largely governed by the number of the already adsorbed surfactants; in other words, we use the approximation ρ ≈ φ in the construction of *G*_attr_. This approximative approach will avoid heavy calculations without compromising on the physical conclusions because the conformations of polymer chains are network-like in semidilute and concentrated polymer solutions. Notice that a physical boundary condition is explicitly embedded in the approximation ρ ≈ φ, that is, both φ → 0 and φ → 1 leading to the vanishing of *G*_attr_ → 0. The approximation correctly reflects the fact that the polyelectrolyte is miscible within aqueous solutions at both low and high concentrations of oppositely charged surfactants in reentrant condensation, respectively.

We note that previous experiments^66–68^ reported that the accumulation or preferential adsorption of surfactant ions on polyelectrolyte chains can lead to charge inversion of some polyelectrolytes at certain concentrations of oppositely charged surfactants. This implies in physics that the free energy of the polyelectrolyte solution must have a minimum (or minimum) or exhibit symmetry breaking around these surfactant concentrations. Notice that this consideration is reflected by the statistical construction of *G*_attr_ in an interpolative way with a minimum and a symmetry breaking around φ = 2/3 where the coupling factor φ^2^(1 – φ) shows its maximum, which corresponds to the fact that the symmetry of simple adsorption and desorption states of ionic monomers (φ or 1 – φ states) by surfactant ions is broken and which further corresponds to the maximum coupling between adsorption and attraction effects of surfactant ions on polyelectrolyte chains when the phase transition occurs from a soluble to a collapsed state or vice versa (including both collapse and reentry transitions). This viewpoint will become clear in detail in the analytical analyses in Sections 3.1 and 3.2. Because of this feature, the fraction of polyelectrolyte chains adsorbed preferentially by surfactant ions (φ) is also the order parameter for the phase transition in our model.

The surfactant concentration at which the reentrant condensation of the polyelectrolyte occurs is usually below about 100 mmol/L.^17–20^ This fact indicates that the free energy of nonassociative pairwise-like electrostatic interaction due to the long-range correlation of all ions can be approximated by the classical “double screening theory” ^22,69^ in the low-salt limit as *G*_DS_ with its truncated form, see the fifth line of eq 1. Here, the nonassociative attraction correlation of all small ions is approximated by the Debye–Hückel theory^70^ for the isothermal–isobaric (*NPT*) ensemble. The inverse Debye screening length κ is given by (κ*a*)^2^ = 4π­(*l*_B_/*a*)­(*pc* + (*n* + 1)*c*_*x*_) for polyelectrolytes in diluted surfactant solutions, where *l*_B_ is the Bjerrum length of water. There is a strong charge neutralization effect^10,15^ when monovalent surfactant ions adsorb on monovalent ionic monomers because of the formation of electrostatic dipoles. This results in the contribution of the electrostatic repulsion between ionized monomers for *G*_DS_ being insignificant compared with other terms in free energy because the charge–charge interaction between ionized monomers is highly screened (see a similar argument in chapter six of the excellent monograph by Muthukumar^21^). Therefore, in the following discussion, we will ignore it to simplify analytical calculations.

Then, the total Gibbs free energy per volume unit is simply considered as *G*(φ, *c*, *c*_*x*_) = *G*_sol_ + *G*_FH_ + *G*_ads_ + *G*_attr_ + *G*_DS_. The essential idea of our model is that the short-range hydrophobic-aggregation effect of a polyelectrolyte solution is statistically constructed by the key formalism of *G*_attr_ in eq 1. And the long-range electrostatic correlation of all ions is considered by the classical “double screening theory”,^22,69^ i.e., *G*_DS_ in eq 1. We note that this approach for long-range electrostatic interactions does not require that the polyelectrolyte has to be dilute, as indicated by a recent comprehensive study.^71^ We list the symbols used in the theory in Table 1 for the reader’s convenience. Notice that in this study, we deliberately neglect the possible hydrophobic adsorption between the surfactant tail and a hydrophobic polymer backbone, as well as the related electrostatic repulsion effect among adsorbed surfactant ions. This approach will significantly simplify our analytical calculations and guide us to focus on the well-studied phase-transition mechanism,^20,25–28^ as illustrated by Figure 1b, and will finally lead us to qualitative but rather simple answers for the two fundamental questions raised in the Introduction.

**Table 1:** List of Symbols Used in the Theory

symbols	physical meaning of the symbol
*N*	the number of monomers in a polyelectrolyte chain
*n*	the surfactant chain size in units of a polyelectrolyte monomer
*a*	the size of a monomer along the direction of the polymer backbone, the size of a solvent molecule, the size of a surfactant counterion, and the size of a polyelectrolyte counterion
*p*	the fraction of charged monomers in a polyelectrolyte chain
∏	the osmotic pressure acting on the polymer phase by the bulk
*c*	the volume fraction of monomers
*c*_*x*_	the volume fraction of counterion of a surfactant
*k*_B_	the Boltzmann constant
*T*	the thermodynamic temperature
φ	the fraction of ionic monomers occupied preferentially by the surfactant ions
λ ≈ 1	the volume ratio between the ionic head of a surfactant ion and a solvent (or a counterion)
χ_s_	the parameter considering the demixing effect between a surfactant and water on polyelectrolyte chains
μ	the exchange chemical potential for a surfactant ion on the polymer chains
*nc*_*x*_ ≪ ρ → φ	the volume fraction of the surfactant ion next to polyelectrolyte chains in the solvent phase
*l*_B_	the Bjerrum length of water
κ	the inverse Debye screening length
ε_1_	the excess-adsorption strength of one surfactant ion with respect to the monovalent counterion of the polyelectrolyte on its ionic monomers
ε_2_	the hydrophobic-aggregation strength among surfactant ions that are adsorbed on ionic monomers
ε_FH,1_	the Flory–Huggins parameter between the solvent and charged monomers
ε_FH,2_	the Flory–Huggins parameter between the solvent and uncharged monomers
*G*_sol_	the free energy owing to the mixing of the polymer with solvent and added surfactant
*G*_ads_	the adsorption free energy owing to the mixing of surfactant ions and polyelectrolyte counterions on the polymer chains
*G*_attr_	the associative attraction between ionized monomers caused by a temporary bridge due to the hydrophobic-aggregation effect of adsorbed surfactant ions on ionic monomers
*G*_DS_	the free energy of nonassociative pairwise-like electrostatic interaction due to the *long-range* correlation of all ions in the low-salt limit
*G*_FH_	the energy of nonelectrostatic excluded-volume interactions between the solvent (water) and the charge-neutral part of monomers
*G*(φ, *c*, *c*_*x*_)	the total Gibbs free energy per volume unit under the framework of the isothermal–isobaric (*NPT*) ensemble

## 3 Results and Discussion

### 3.1 The Minimum Coupling Energy for the Hydrophobic-Aggregation Effect in the Phase Transition

Following a similar computing approach as our previous work^72^ and the work by Sommer,^73^ we can estimate a minimum coupling energy (ε_2_) for the hydrophobic-aggregation effect that is necessary for a phase transition. Based on the construction of *G*_attr_ in eq 1, we see that the maximum contribution to the adsorption and attraction effects of surfactant ions on polyelectrolyte chains occurs around the symmetry-breaking point φ = 2/3. As long as this contribution is dominant so that it defines the overall symmetry properties of the entire free energy function, this simplification allows us to mathematically cast our model to a canonical ensemble-like model by setting φ = 2/3 for the case of a very diluted solution of the surfactant ((*n* + 1)*c*_*x*_ → 0), at which both the chemical potential μ and the osmotic pressure ∏ approach their limiting values μ_0_ and ∏_0_, respectively:

2 $$\begin{array}{l} G-\Pi_{0}=\frac{c}{N}\ln\left(c\right)+pc\;\ln\left(pc\right)+\left[1-\left(1+p\right)c\right]\ln\left[1-\left(1+p\right)c\right]\\ +\left[\frac{2\;\ln2}{3\lambda }-\left(\frac{2}{3\lambda }+\frac{1}{3}\right)\ln3-\frac{2}{3}\left(\mu_{0}+\frac{\varepsilon_{1}}{\lambda }\right)+\frac{2}{9}\chi_{s}\right]pc\\ -\frac{4}{27}\varepsilon_{2}{\left(pc\right)}^{2}-\sqrt{\pi }{\left(\frac{l_{B}}{a}pc\right)}^{3/2}\\ +\left[1-\left(1+p\right)c\right]\left[p\varepsilon_{FH,1}+\left(1-p\right)\varepsilon_{FH,2}\right]c \end{array}$$

Here, the canonical ensemble-like free energy is given by *G* – ∏_0_.

To show a noticeable hydrophobic effect, the surfactant chain length must be above a minimum length. This implies that there exists a minimum coupling energy for the hydrophobic-aggregation effect (ε_2_) that is necessary for a phase transition. By eq 2, we can roughly estimate a minimum value for the coupling energy (ε_2_). This can be realized by determining the boundary condition for the spinodal decomposition of polyelectrolyte solution, which is given by d^2^(*G* – ∏_0_)/d*c* ^2^ = 0 in analogy to the framework of the canonical ensemble and follows with refs 72 and 73

3 $$\begin{array}{l} 0=\frac{d^{2}\left(G-\Pi_{0}\right)}{dc^{2}}=-\frac{8}{27}\varepsilon_{2}p^{2}-2\varepsilon_{FH,1}\left(1+p\right)p-2\varepsilon_{FH,2}\left(1-p^{2}\right)\\ +\frac{1}{Nc}+\frac{p}{c}+\frac{{\left(1+p\right)}^{2}}{1-\left(1+p\right)c}-\frac{3\sqrt{\pi }}{4}{\left(\frac{l_{B}}{a}p\right)}^{3/2}c^{-1/2} \end{array}$$

Here, we define an overall effective interaction parameter 2χ_0_ ≡ 8ε_2_ *p* ^2^/27 + 2ε_FH,1_(1 + *p*)*p* + 2ε_FH,2_(1 – *p* ^2^). Its critical or minimum value to allow phase transition is given by d­(*2*χ_0_)/d*c* = 0, which follows the approach by classical monographs.^74,75^ This approach is mathematically equivalent to d^3^(*G* – ∏_0_)/d*c* ^3^ = 0, which^76^ finds the critical point for the free energy *G* – ∏_0_,

4 $$0=\frac{d\left(2\chi_{0}\right)}{dc}=-\frac{1}{Nc^{2}}-\frac{p}{c^{2}}+\frac{{\left(1+p\right)}^{3}}{{\left[1-\left(1+p\right)c\right]}^{2}}+\frac{3\sqrt{\pi }}{8}{\left(\frac{l_{B}}{a}\frac{p}{c}\right)}^{3/2}$$

There exists no exact explicit analytical solution for *c* in eq 4, but an acceptable approximation can be obtained by ignoring the fourth term since it is much smaller than the sum of the other terms within experimental values of the parameter *l*_B_/*a*. For the critical point of the polyelectrolyte collapse, we obtain the approximation for the critical volume fraction of monomers *c* at

5 $$\frac{1}{c}≃\left(1+p\right)+\frac{{\left(1+p\right)}^{3/2}}{{\left(\frac{1}{N}+p\right)}^{1/2}}$$

The exact solution of eq 4 is recovered by eq 5 when the parameter *p* approaches zero. One can improve upon the analytical solution of eq 4 by employing the method of fixed-point iteration^77–79^ with the initial value defined by eq 5. However, the approximative solution of eq 5 is sufficient to deduce the key features of the overall effective interaction parameter χ_0_ without compromising the physical conclusions. The details behind the computational solution of this approach can be found in Section A of Supporting Information.

By insertion of eq 5 into eq 3, we get an estimation of the critical or minimum value of χ_0_,

6 $$\begin{array}{l} 2\chi_{0,\min}\equiv \frac{8}{27}\varepsilon_{2}p^{2}+2\varepsilon_{FH,1}\left(1+p\right)p+2\varepsilon_{FH,2}\left(1-p^{2}\right)\\ ≃{\left[{\left(\frac{1}{N}+p\right)}^{1/2}{\left(1+p\right)}^{1/2}+\left(1+p\right)\right]}^{2}-\frac{3\sqrt{\pi }}{4}{\left(\frac{l_{B}}{a}p\right)}^{3/2}{\left[\left(1+p\right)+\frac{{\left(1+p\right)}^{3/2}}{{\left(\frac{1}{N}+p\right)}^{1/2}}\right]}^{1/2} \end{array}$$

Equation 6 shows that a shorter polymer chain (*N*) prescribes a higher minimum coupling energy (ε_2_) that is necessary for a phase transition. This correctly reflects the obvious but nontrivial fact that a shorter polymer chain requires a higher enthalpic attraction among polymer chains to overcome/compensate for a larger translational entropy of polymer chains in phase transition. By setting *p* = 0 in eq 6, we recover the boundary condition for the spinodal decomposition of an uncharged polymer solution, i.e., $\varepsilon_{FH,2}=\frac{1}{2}{\left(1+\frac{1}{\sqrt{N}}\right)}^{2}$ . We derive the minimum of ε_2_ for the case of a very long polyelectrolyte chain (*N* → ∞) by eq 6,

7 $$\begin{array}{l} {\left(\varepsilon_{2}\right)}_{\min}≃\frac{27}{8}{\left[{\left(\frac{1+p}{p}\right)}^{1/2}+\frac{1+p}{p}\right]}^{2}-\frac{27\left[\varepsilon_{FH,1}\left(1+p\right)p+\varepsilon_{FH,2}\left(1-p^{2}\right)\right]}{4p^{2}}\\ -\frac{81\sqrt{\pi }}{32}{\left(\frac{l_{B}}{a}\right)}^{3/2}{\left[\frac{1+p}{p}+{\left(\frac{1+p}{p}\right)}^{3/2}\right]}^{1/2} \end{array}$$

As discussed before, the strength of the hydrophobic-aggregation effect obviously depends on the surfactant’s chain length. It is also well-known that the strength of hydrophobic attraction is about 1.5 *k*_B_ *T* between two methyl/methylene groups.^80^ Therefore, there is a minimum surfactant chain length (*n*_min_)^55–58^ to induce phase separation of the polyelectrolyte, which is numerically estimated by *n*_min_ ≈ 2­(ε_2_)_min_/3 according to eqs 6 and 7. As an example of a hydrophilic polyelectrolyte, we choose model parameters *p* ≈ 0.9, *l*_B_/*a* ≈ 2, *N* = 6000 (or *N* = 30), and ε_FH,1_ = ε_FH,2_ ≈ 0.41 for ionized poly­(acrylic acid);^81,82^ then, eq 6 predicts that the minimum surfactant chain length to induce phase separation is about *n*_min_ ≈ 7 for the mixtures of ionized poly­(acrylic acid) and alkyl trimethylammonium bromides. It is noticeable that this prediction is quite close to its experimental value (*n*_min_ ≈ 8)^56^ for the chosen set of model parameters. For the coacervation of another polyelectrolyte poly­(diallyl dimethylammonium chloride) with surfactant sodium alkyl sulfate,^55^ one can estimate a similar minimum surfactant chain length (*n*_min_ ≈ 8) to initiate phase transition by using similar experimental values of model parameters.^83^

A feature of the constructions of eqs 6 and 7 is that the minimum coupling energy (ε_2_)_min_ for the hydrophobic-aggregation effect estimated by eqs 6 and 7 is not related to the model parameters ε_1_, λ, and χ_s_. This is an expected result because one cannot expect a phase transition merely according to the simple exchange effect of surfactant ions on polymer chains based on a formal analogy with the well-understood 1D-Ising model.^84,85^ Here, if we solely take into account pairwise-like interactions for preferential adsorption between surfactant ions and ionic monomers, linear polymers are 1D substrates for ions.

Another feature of the constructions of eqs 6 and 7 is that, as shown in Figure 2, no matter the values of *l*_B_/*a* ≥ 0 and ε_FH,1_ ≥ 0, there is a unique local maximum of (ε_2_)_min_ when ε_FH,2_ is larger than $\frac{1}{2}{\left(1+\frac{1}{\sqrt{N}}\right)}^{2}$ , but no local maximum of (ε_2_)_min_ exists when 0 ≤ ε_FH,2_ ≤ $\frac{1}{2}{\left(1+\frac{1}{\sqrt{N}}\right)}^{2}$ . This feature is coincidental with the well-known Θ condition for uncharged polymers. Thus, we can see that the minimum coupling energy (ε_2_)_min_ for the hydrophobic-aggregation effect to lead to a phase transition is related to the solvent quality (parameter ε_FH,2_) for the uncharged part of the polyelectrolyte. We note that this analytical result reflects the experimental fact^20,39^ that the hydrophobicity of uncharged monomers of polyelectrolytes has a noticeable effect on the necessary surfactant concentration to trigger the reentry transition.

**Figure 2:**
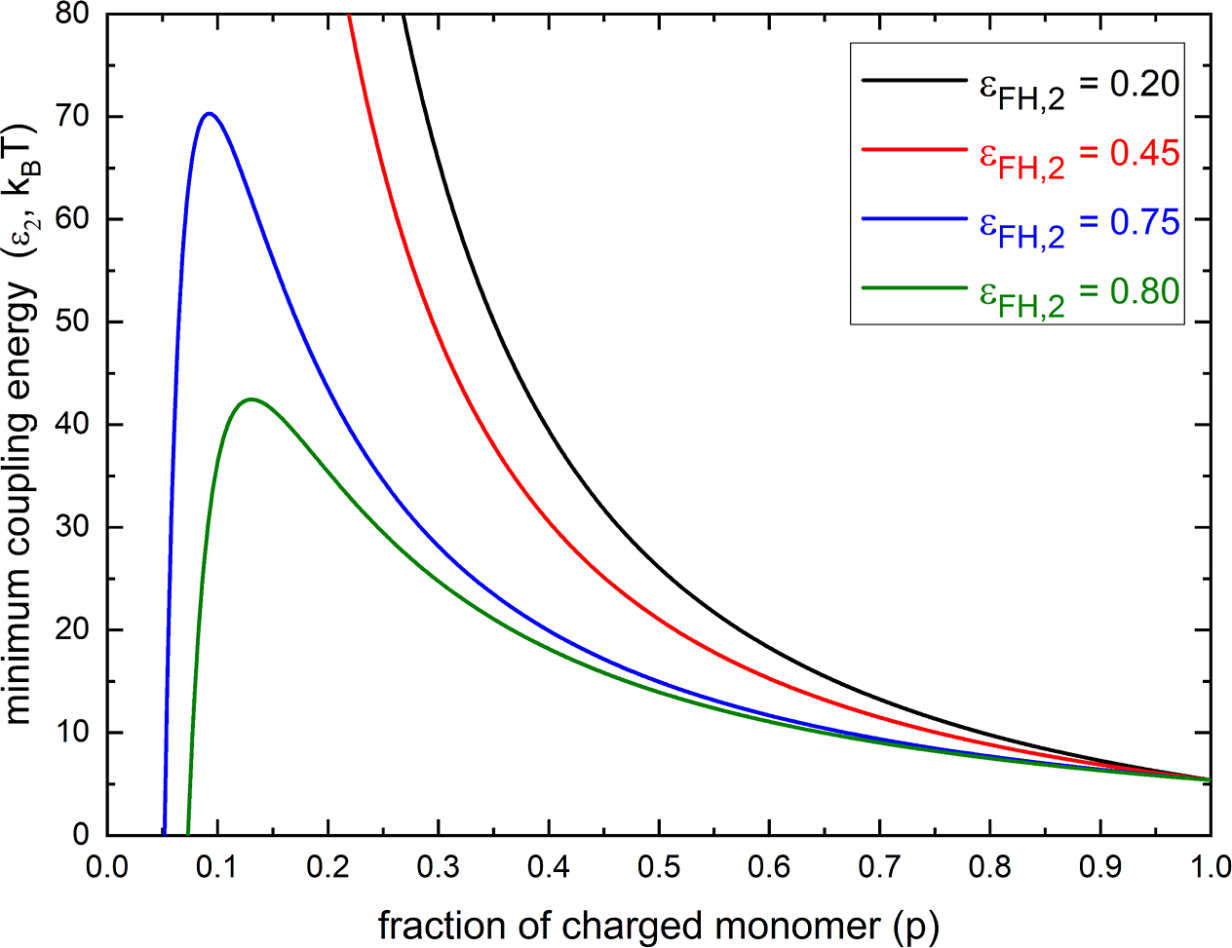
According to eq 7, the minimum coupling energy (ε_2_) with respect to the fraction of charged monomer (*p*) for typical values of the parameter ε_FH,2_ with *l*_B_/*a* = 2, ε_FH,1_ = 0.45, and *N* → ∞.

### 3.2 The Nonmonotonic Effective Flory–Huggins χ Parameter

In order to find the equilibrium state of the polyelectrolyte phase with respect to the bulk solvent phase, we follow a similar computing approach as in our previous work.^72^ First, we minimize the free energy *G*(φ, *c*, *c*_*x*_) with respect to the adsorption fraction of surfactant ions φ. Then, the replacement of the equilibrium solution for φ­(μ, *c*, *c*_*x*_) into the free energy leads to an effective free energy for the polyelectrolyte, in which the effect of surfactant ions is mapped onto an effective monomer–monomer interaction, which depends on the concentration of surfactant ions. Finally, the minimization of the free energy per monomer, i.e., *G*(φ, *c*, *c*_*x*_)/*c* instead of *G*(φ, *c*, *c*_*x*_), with respect to the monomer concentration (*c*) leads to the equilibrium solution of the model.

Because we are interested in the case of a very diluted solution of surfactant ((*n* + 1)*c*_*x*_ → 0) in this work, we ignore the influence of surfactant concentration in the bulk solution in the construction of *G*_sol_, *G*_FH_, and *G*_DS_. The physical foundation behind this assumption is that the surfactant concentration for the reentrant condensation of a polyelectrolyte can be several orders of magnitude below the bulk critical micelle concentration (CMC) of the surfactant,^24^ which implies that the influence of mixing behaviors of the surfactant in bulk solution is negligible for the reentrant condensation. This assumption allows us to focus on the adsorption and attraction effects of surfactants near/on polymer chains and ignore their own nonessential mixing effects if surfactant molecules are far away from polymer chains, which can avoid heavy calculations without losing generality of the key physical conclusions.

The construction of *G*_attr_ in eq 1 implies that the maximum contraction of polymer chains is reached around the maximum-coupling point φ = 2/3, where the hydrophobic-aggregation effect reaches its maximum, which corresponds to the phase transition from a soluble to a collapsed state or vice versa (including both collapse and reentry transitions). This peculiar feature of the model leads us to the introduction of a perturbation (δ) from the maximum-coupling point φ = 2/3 according to

8 $$\varphi =\frac{2}{3}\left(1-\delta \right)$$

This perturbation approach follows closely our previous work.^86^ With the Taylor expansion of δ-containing terms in the logarithm function up to the accuracy of square terms (δ^2^) under the constraint of |δ^3^| ≪ 1 and ignoring constant terms, we obtain

9 $$\begin{array}{l} \frac{G\left(\delta ,c\right)}{c}=\left(\frac{G_{sol}+G_{DS}+G_{FH}}{c}\right)+\frac{2}{3}\left(\mu +\frac{\varepsilon_{1}+\ln3-\ln2}{\lambda }-\ln3\right)p\delta \\ +\frac{2}{3}\left(1-\frac{1}{\lambda }\right)p\delta +\frac{1}{3}\left(2+\frac{1}{\lambda }\right)p\delta^{2}+\frac{2}{9}\chi_{s}\left(\delta -2\delta^{2}\right)p-\frac{4}{27}\varepsilon_{2}\left(1-3\delta^{2}\right)p^{2}c \end{array}$$

with

10 $$\begin{array}{l} \frac{G_{sol}+G_{DS}+G_{FH}}{c}=\frac{\ln\left(c\right)}{N}+p\;\ln\left(pc\right)+\left[\frac{1}{c}-\left(1+p\right)\right]\ln\left[1-\left(1+p\right)c\right]\\ +\left[1-\left(1+p\right)c\right]\left[p\varepsilon_{FH,1}+\left(1-p\right)\varepsilon_{FH,2}\right]-\sqrt{\pi }{\left(\frac{l_{B}}{a}p\right)}^{3/2}\sqrt{c}+\frac{\Pi }{c} \end{array}$$

The numerical term “ln 2” in eq 9 is due to the fact that the symmetry of simple adsorption and desorption states of ionic monomers (φ or 1 – φ states) by surfactant ions is broken. This can be seen from the transformation of *G*_ads_ in eq 1 by the manipulation of eq 8.

Minimizing the free energy in eq 9 with respect to δ yields

11 $$\delta =-\frac{3\left(\mu +\frac{\varepsilon_{1}+\ln3-\ln2}{\lambda }-\ln3\right)+\chi_{s}+3\left(1-\frac{1}{\lambda }\right)}{4\varepsilon_{2}pc+3\left(2+\frac{1}{\lambda }\right)-4\chi_{s}}$$

When the surfactant’s bulk chemical potential μ is close to μ_C_ = −((ε_1_ + ln 3 – ln 2)/λ – ln 3 + χ_s_/3 + 1 – 1/λ), the value of δ is close to zero. This case ideally follows the small perturbation expansion introduced by eq 8 and is also the most important situation we are going to focus on in this research. This is discussed in further detail in the following subsections. Resubstituting eq 11 into eq 9, yields

12 $$\begin{array}{l} \frac{G\left(c\right)}{c}=-\frac{4}{27}\varepsilon_{2}p^{2}c-\frac{p{\left[\left(\mu +\frac{\varepsilon_{1}+\ln3-\ln2}{\lambda }-\ln3\right)+\frac{\chi_{s}}{3}+\left(1-\frac{1}{\lambda }\right)\right]}^{2}}{4\varepsilon_{2}pc+3\left(2+\frac{1}{\lambda }\right)-4\chi_{s}}+\left(\frac{G_{sol}+G_{DS}+G_{FH}}{c}\right)\\ =p\varepsilon_{FH,1}+\left(1-p\right)\varepsilon_{FH,2}+p\;\ln\left(p\right)-\frac{p{\left[\left(\mu +\frac{\varepsilon_{1}+\ln3-\ln2}{\lambda }-\ln3\right)+\frac{\chi_{s}}{3}+\left(1-\frac{1}{\lambda }\right)\right]}^{2}}{3\left(2+\frac{1}{\lambda }\right)-4\chi_{s}}\\ -\chi_{eff}c+g_{sol} \end{array}$$

with the effective Flory–Huggins parameter χ_eff_ as follows:

13 $$\begin{array}{l} \chi_{eff}=\varepsilon_{FH,1}\left(1+p\right)p+\varepsilon_{FH,2}\left(1-p^{2}\right)+\frac{4}{27}\varepsilon_{2}p^{2}\\ -\left(4\varepsilon_{2}p^{2}\right)\frac{{\left[\left(\mu +\frac{\varepsilon_{1}+\ln3-\ln2}{\lambda }-\ln3\right)+\frac{\chi_{s}}{3}+\left(1-\frac{1}{\lambda }\right)\right]}^{2}}{\left[4\varepsilon_{2}pc+3\left(2+\frac{1}{\lambda }\right)-4\chi_{s}\right]\left[3\left(2+\frac{1}{\lambda }\right)-4\chi_{s}\right]}+\sqrt{\pi }{\left(\frac{l_{B}}{a}p\right)}^{3/2}\frac{1}{\sqrt{c}} \end{array}$$

and the entropic term of free energy per monomer *g*_sol_ as follows:

14 $$g_{sol}=\frac{\ln\left(c\right)}{N}+p\;\ln\left(c\right)+\left[\frac{1}{c}-\left(1+p\right)\right]\ln\left[1-\left(1+p\right)c\right]+\frac{\Pi }{c}$$

The above constructed χ-function is a function of the square of the chemical potential μ, which indicates that a χ_eff_ corresponds to two values of μ and thus captures the reentrant signature of polyelectrolyte condensation at lower and higher surfactant concentrations. According to eq 13, we know that the reentrant signature of polyelectrolyte condensation is controlled by the hydrophobic-aggregation effect, since only this effect is nonmonotonic with respect to the surfactant concentration.

### 3.3 The General Spinodal and Binodal Phase Diagrams

In order to analytically discuss the phase transition, the pressure isotherm Π­(*c*, μ; *N*, *l*_B_/*a*, *p*, ε_1_, ε_2_, ε_FH,1_, ε_FH,2_) can be calculated from eq 12 by the condition ∂(*G/c*)/∂*c* = 0, which leads to

15 $$\begin{array}{l} \Pi =4\varepsilon_{2}p^{2}c^{2}\left\{\frac{{\left[\left(\mu +\frac{\varepsilon_{1}+\ln3-\ln2}{\lambda }-\ln3\right)+\frac{\chi_{s}}{3}+\left(1-\frac{1}{\lambda }\right)\right]}^{2}}{{\left[4\varepsilon_{2}pc+3\left(2+\frac{1}{\lambda }\right)-4\chi_{s}\right]}^{2}}-\frac{1}{27}\right\}+\left(\frac{1}{N}-1\right)c\\ -\left[p\left(1+p\right)\varepsilon_{FH,1}+\left(1-p^{2}\right)\varepsilon_{FH,2}\right]c^{2}-\frac{\sqrt{\pi }}{2}{\left(\frac{l_{B}}{a}pc\right)}^{3/2}-\ln\left[1-\left(1+p\right)c\right] \end{array}$$

By setting *p* = 0 in eq 15 for an uncharged polymer solution, with ∂Π/∂c = 0 and the constraint of 0 < *c* < 1, as it should be, we recover the boundary condition for the spinodal decomposition of the uncharged polymer solution, i.e., $\varepsilon_{FH,2}>\frac{1}{2}{\left(1+\frac{1}{\sqrt{N}}\right)}^{2}$ .

For the general case of a discontinuous phase transition, the osmotic pressure must display an unstable region of negative compressibility given by ∂Π/∂*c* < 0. In Figure 3a, we display two examples showing the two critical points of the pressure isotherms given by eq 15, i.e., corresponding to the states of ∂Π/∂c = ∂^2^Π/∂c^2^ = 0. In Figure 3b, we display an example that shows the pressure isotherms for a discontinuous condensation transition given by eq 15. The coexistence region of the binodal is defined by the Maxwell construction as indicated by the equal area criterion (the area of the envelope “ABC” is equal to that of the envelope “CDE”: *S*_1_ = *S*_2_) with the horizontal isobaric line in the figure (the black dashed line “ACE”). This cannot be obtained analytically in an exact way but must be computed by numerical calculations. However, we are able to calculate the spinodal state analytically at which the solution begins to turn unstable, the existence of which is the necessary condition for a discontinuous transition scenario. The spinodal of the polyelectrolyte solution is given by ∂Π/∂c = 0 and can be written in the following form:

16 $$U^{2}=\frac{{\left[4\varepsilon_{2}pc+3\left(2+\frac{1}{\lambda }\right)-4\chi_{s}\right]}^{3}}{8p^{2}\varepsilon_{2}c\left[3\left(2+\frac{1}{\lambda }\right)-4\chi_{s}\right]}\left\{\begin{array}{l} \frac{8\varepsilon_{2}p^{2}}{27}c+2\left[p\left(1+p\right)\varepsilon_{FH,1}+\left(1-p^{2}\right)\varepsilon_{FH,2}\right]c\\ +\frac{3\sqrt{\pi }}{4}{\left(\frac{l_{B}}{a}p\right)}^{3/2}\sqrt{c}-\frac{\left(1+p\right)}{1-\left(1+p\right)c}+\left(1-\frac{1}{N}\right) \end{array}\right\}$$

Here, we denote the external adsorption field *U* as

17 $$U\equiv \left(\mu +\frac{\varepsilon_{1}+\ln3-\ln2}{\lambda }-\ln3\right)+\frac{\chi_{s}}{3}+\left(1-\frac{1}{\lambda }\right)$$

This defines the spinodal phase diagram in the “μ—*c*” space with the eight parameters *p*, *N*, *l*_B_/*a*, ε_1_, ε_2_, χ_s_, ε_FH,1_, and ε_FH,2_.

**Figure 3:**
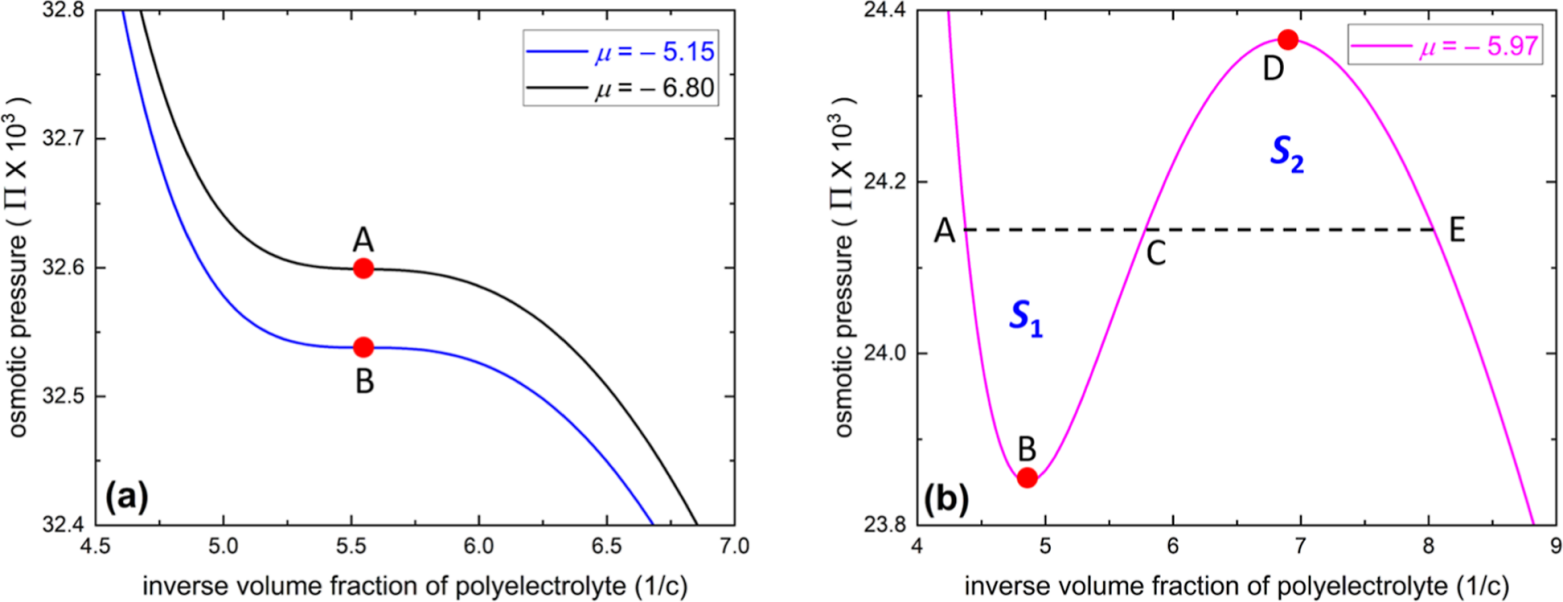
According to eq 15, the osmotic pressure of the polyelectrolyte solution is plotted as a function of the inverse volume fraction of polyelectrolyte monomers for the parameters *p* = 0.6, *l*_B_/*a* = 2, ε_1_ = 6, ε_2_ = 15, ε_FH,1_ = 0.4, ε_FH,2_ = 0.4, λ = 1, χ_s_ = 2, and *N* → ∞. In panel (a), we show the two critical points (indicated by filled circles with symbols “A” and “B”) of the pressure isotherms by choosing μ = −6.80 and μ = −5.15, which also correspond to μ_A_ and μ_B_, respectively, in Figure 4. In panel (b), we choose μ = −((ε_1_ + ln 3 – ln 2)/λ – ln 3 + χ_s_/3 + 1 – 1/λ) = −5.97, which also corresponds to μ_C_ in Figure 4. The coexistence pressure of the binodal, obtained by the Maxwell construction, is indicated by the horizontal dashed line in the figure (the black dashed line “ACE”) with the equal area criterion (the area of the envelope “ABC” is equal to that of the envelope “CDE”: *S*_1_ = *S*_2_), and the spinodal points are indicated by filled circles in the figure (symbols “B” and “D”).

In Figure 4, we display the spinodal phase diagram (closed red curve “ACBD”) in the “μ—*c*” space given by eq 16 and its corresponding binodal diagram (closed blue curve “AEBF”) by numerical calculations for a typical case of polyelectrolyte with parameters *p* = 0.6, *l*_B_/*a* = 2, ε_1_ = 6, ε_2_ = 15, ε_FH,1_ = ε_FH,2_ = 0.4, λ = 1, χ_s_ = 2, and *N* → ∞. The two solutions at the same value of μ correspond to the two extrema of the pressure isotherm, which imply the coexistence of a condensed polymer phase and a dissolved polymer phase. The region of phase separation is closed topologically. For our theory, we obtain collapse and re-entry transitions that are symmetric; for example, the closed curves in Figure 4 are symmetric with respect to the tie-line “ECDF”. The lower part of μ defines the collapse transition as indicated by the lower half of the “egg-shape” curve, while the higher part of μ defines the reentry transition. In Figure 4, μ_A_ indicates the surfactant concentration above which the collapse transition can occur, which is close to the critical aggregation concentration (CAC). μ_B_ indicates the surfactant concentration below which the reentry transition can take place, which is close to the second critical aggregation concentration (CAC_2_). μ_C_ indicates the surfactant concentration at which the polyelectrolyte solution shows the maximum extent of phase separation and the effective Flory–Huggins parameter χ_eff_ reaches its maximum (see eq 13).

**Figure 4:**
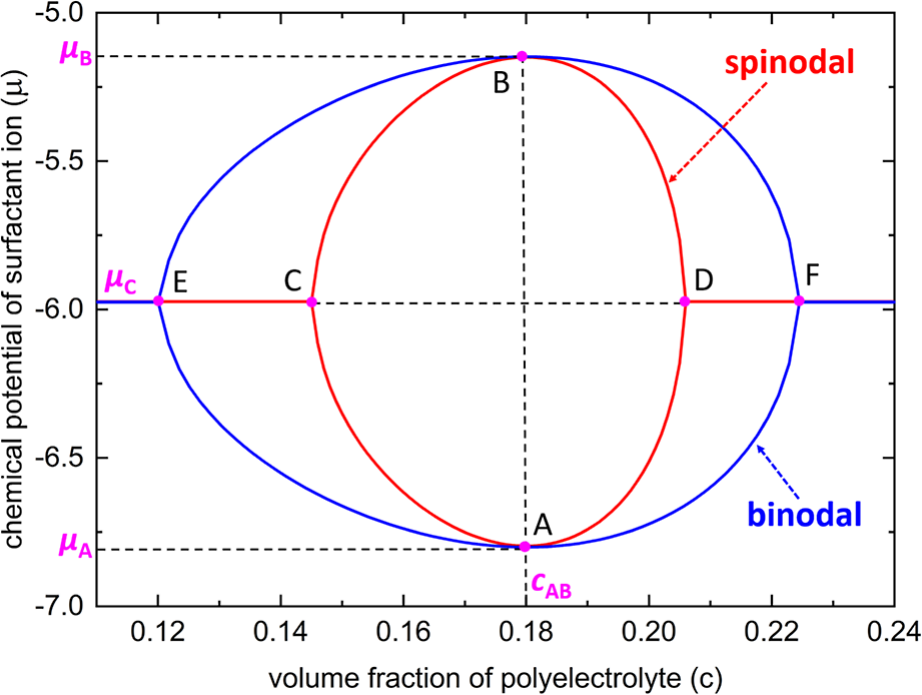
According to eq 16, a typical spinodal phase diagram (closed red curve “ACBD”) in the “μ—*c*” space and its corresponding binodal diagram (closed blue curve “AEBF”), obtained by numerical calculations for a polyelectrolyte in the dilute solution of an oppositely charged surfactant for the case of *p* = 0.6, *l*_B_/*a* = 2, ε_1_ = 6, ε_2_ = 15, ε_FH,1_ = ε_FH,2_ = 0.4, λ = 1, χ_s_ = 2, and *N* → ∞. Both the binodal diagram and the spinodal diagram are symmetric with respect to the tie-line “ECDF”. In the figure, μ_A_ indicates the lower limit surfactant chemical potential for the collapse transition, μ_B_ indicates the upper limit surfactant chemical potential for the reentry transition, and μ_C_ indicates the chemical potential at which the polyelectrolyte solution shows the maximum extent of phase separation.

From Figure 4, we see that the external adsorption field *U* has a minimum and a maximum at μ_A_ and μ_B_, respectively. Because we obtain symmetric collapse and reentry transitions for our theory, these facts indicate that there is a unique monomer concentration (*c*_AB_) corresponding to both μ_A_ and μ_B_. It can be solved in a formal way by

18 $$0=\frac{\partial \left(U^{2}\right)}{\partial c}$$

Then, the values of μ_A_ and μ_B_ can be obtained by the insertion of the unique solution of the monomer concentration (*c*_AB_) into eq 16. It is remarkable that the unique monomer concentration (*c*_AB_) at both μ_A_ and μ_B_ are also the two critical points of the osmotic pressure in eq 15, i.e., corresponding to the solution of ∂Π/∂c = ∂^2^Π/∂c^2^ = 0. As shown in Figure 4, this property can be seen graphically since there are two symmetric tangent points, and each is at (*c*_AB_, μ_A_) and (*c*_AB_, μ_B_) for the spinodal and its corresponding binodal phase diagrams. Here, we point out that in general, the best way to obtain μ_A_ and μ_B_ is via numerical methods because it is challenging to derive simple analytical approximations. Nevertheless, because we obtain symmetric collapse and reentry transitions for our theory, we can derive the following exact and simple relations:

19 $$\begin{array}{l} \mu_{C}=\frac{\mu_{A}+\mu_{B}}{2}\\ 0=U\equiv \left(\mu_{C}+\frac{\varepsilon_{1}+\ln3-\ln2}{\lambda }-\ln3\right)+\frac{\chi_{s}}{3}+\left(1-\frac{1}{\lambda }\right) \end{array}$$

The values of μ_A_, μ_B_, and μ_C_ are usually comparable with each other in experimental observations;^20,25–28^ this can also be easily seen from the example in Figure 4.

As shown in Figure 4, when the bulk chemical potential of surfactant ions is close to μ_C_, the tie-line “ECDF” shows that there is an obvious difference in the polymer concentration of the dilute polymer phase as predicated by the spinodal and binodal phase diagrams (symbols “C” and “E” in Figure 4). A similar noticeable difference is also observed for the polymer concentration of the condensed polymer phase (symbols “D” and “F” in Figure 4). This basic characteristic of our model implies that there may exist rich nonequilibrium/metastable/kinetically trapped states when the polymer–surfactant coacervates evolve from a spinodal to a binodal state—considering the fact that the time scales to reach a true equilibrium state for a polymer solution can span months or even years,^87^ which were indeed observed in experiments.^8,9,11^

Another basic characteristic of our model is that it predicts that complete polyelectrolyte compaction can occur at far less than 100% of the degree of charge compensation of ionic monomers. This prediction can be easily seen from the tie-line “ECDF” in Figure 4, where the surfactant concentration (μ_C_) is insufficient to compensate for the charges of all ionic monomers when the polyelectrolyte solution shows the maximum extent of phase separation. It is remarkable that this prediction was confirmed in detail by experimental studies^66,67^ on the phase behaviors of both natural and synthetic polyelectrolytes (DNA and poly­(acrylic acid)) in the presence of oppositely charged surfactants. According to eqs 16 and 19, we see that the model shows some flexibility in realizing charge compensations and charge regulations of polyelectrolytes by varying the adsorption strength of surfactant ions on ionic monomers (ε_1_), the demixing strength between the surfactant and water (χ_s_), and the size of the ionic head of a surfactant (λ). The experimental studies^66,67^ and our theory clearly show that it is not necessary to stick to the concept of full charge compensation to explain the collapse transition of a polyelectrolyte in the presence of an oppositely charged surfactant, a concept which was employed by Rumyantsev and co-workers^47^ for the study of polyelectrolyte gels.

### 3.4 Comparison to Additional Experiments

In this subsection, we compare analytical predictions of our theory with additional experimental results reported in the literature.

In Figure 5, we display the spinodal phase diagram according to eq 16 for various polyelectrolyte chain lengths (*N*) under the condition of *p* = 0.6, *l*_B_/*a* = 2, ε_1_ = 6, ε_2_ = 18, ε_FH,1_ = ε_FH,2_ = 0.4, λ = 1, and χ_s_ = 1. As shown in Figure 5, the phase transition can be influenced drastically by the chain length (*N*) of a polyelectrolyte. We see that an increase in polyelectrolyte chain length promotes the coexistence region of collapse transition to a lower surfactant concentration and shifts the coexistence region of reentry transition to a higher surfactant concentration. However, this chain-length dependence diminishes for long polyelectrolyte chains. In contrast to uncharged linear polymer,^21,59,60^ by setting the external adsorption field *U* = 0 in eq 16, our model shows that it is hard to realize a real dilute phase for the reentrant condensation of polyelectrolyte when the charge fraction is not sufficiently low. As indicated in both Figures 4 and 5 for the binodal and spinodal diagrams, this is particularly noticeable in the limiting case of an infinite polyelectrolyte chain length. There remains no small monomer concentration (*c*) in the dilute phase for small values of the external field *U* when phase separation occurs, i.e., close to the optimally loaded state of the polyelectrolyte with surfactant ions where the effective Flory–Huggins parameter χ_eff_ reaches its maximum (see eq 13).

**Figure 5:**
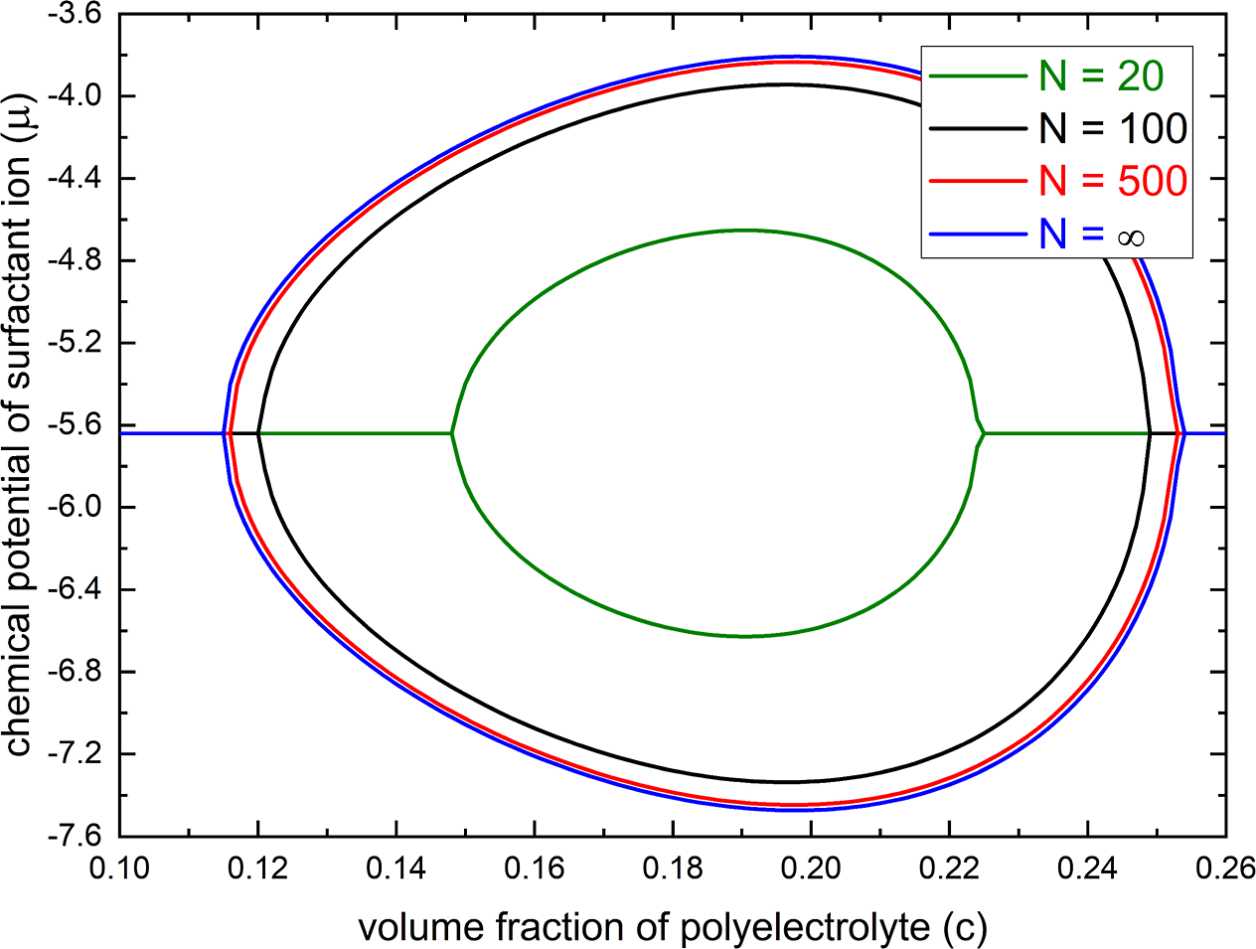
Spinodal phase diagrams of a polyelectrolyte in the dilute solution of an oppositely charged surfactant for different polyelectrolyte chain lengths (*N*) under the condition of *p* = 0.6, *l*_B_/*a* = 2, ε_1_ = 6, ε_2_ = 18, ε_FH,1_ = ε_FH,2_ = 0.4, λ = 1, and χ_s_ = 1, as computed from eq 16.

Our theory also predicts that there is no phase transition if the polyelectrolyte chain length is too short. With the parameter set used for Figure 5, no phase transition occurs with chain lengths of *N* < 10. Actually, this is a consequence of the fact that a shorter polyelectrolyte chain requires a higher minimum coupling energy for the hydrophobic-aggregation effect in phase transition; see eq 6. Furthermore, the impact of translational entropy of the chain increases with shorter chain length, which generally works against the formation of aggregates. It is remarkable that these analytical predictions of our theory were confirmed in detail by previous experimental studies^57,68,88,89^ on the influence of polyelectrolyte chain length in the phase separation of polyelectrolyte admixed with an oppositely charged surfactant.

Remarkably, similar to the above discussion, our theoretical approach can be leveraged to explain and analyze some co-condensation behaviors of DNAs/RNAs with proteins/peptides (such as FUS and HP1 proteins).^29–37^ Here, a relatively short protein–peptide chain acts analogously to a surfactant. For example, the phase separation of DNA induced by protein binding is dependent on the DNA length,^29,35^ which can be explained by our theory on the effect of polyelectrolyte chain length (see discussion for Figure 5). Here, only one specific binding site of a protein anchors on the DNA chains, whereas other nonspecific sections of the protein can interact with other proteins via physical cross-linking, a physical picture which is similar to the phase-transition mechanism shown in Figure 1b.

In Figure 6a, we display the spinodal phase diagram according to eq 16 for different strengths of the hydrophobic-aggregation parameter (ε_2_) under the condition of *p* = 0.6, *l*_B_/*a* = 2, ε_1_ = 6, ε_FH,1_ = ε_FH,2_ = 0.4, λ = 1, χ_s_ = 1, and *N* → ∞. We see that an increase in the strength of the hydrophobic-aggregation effect (ε_2_), which is related to an increase in the surfactant chain length (*n*), shifts the coexistence region of the collapse transition to a lower concentration of the surfactant but shifts the coexistence region of the reentry transition to a higher concentration of the surfactant, which is in agreement with experimental results of highly hydrophilic polyelectrolytes in aqueous solutions of oppositely charged surfactants.^24,55–58^ However, when the surfactant chain length exceeds a maximum length, it is expected that the chain-length effect of the surfactant will saturate due to the lack of an efficient stack of steric surfactant tails in their premicellar aggregation (see Figure 1b for a sketch of the premicellar aggregation). But in this situation, the demixing effect between the surfactant and water (χ_s_) still plays a role on polyelectrolyte chains. In Figure 6b, we display the spinodal phase diagram according to eq 16 for different demixing effects between the surfactant and water (χ_s_) with a parameter set of *p* = 0.6, *l*_B_/*a* = 2, ε_1_ = 6, ε_2_ = 15, ε_FH,1_ = ε_FH,2_ = 0.4, λ = 1, and *N* → ∞. We see that an increase in the demixing effect between the surfactant and water (χ_s_) shifts the coexistence region of both collapse and reentry transitions to lower concentrations of the surfactant. This analytical result indeed qualitatively explains the phase-behavior difference of a highly hydrophilic polyelectrolyte in aqueous solutions of hexadecyl- and dodecyl-trimethylammonium bromides (both surfactants have a very long steric alkyl tail).^20^

**Figure 6:**
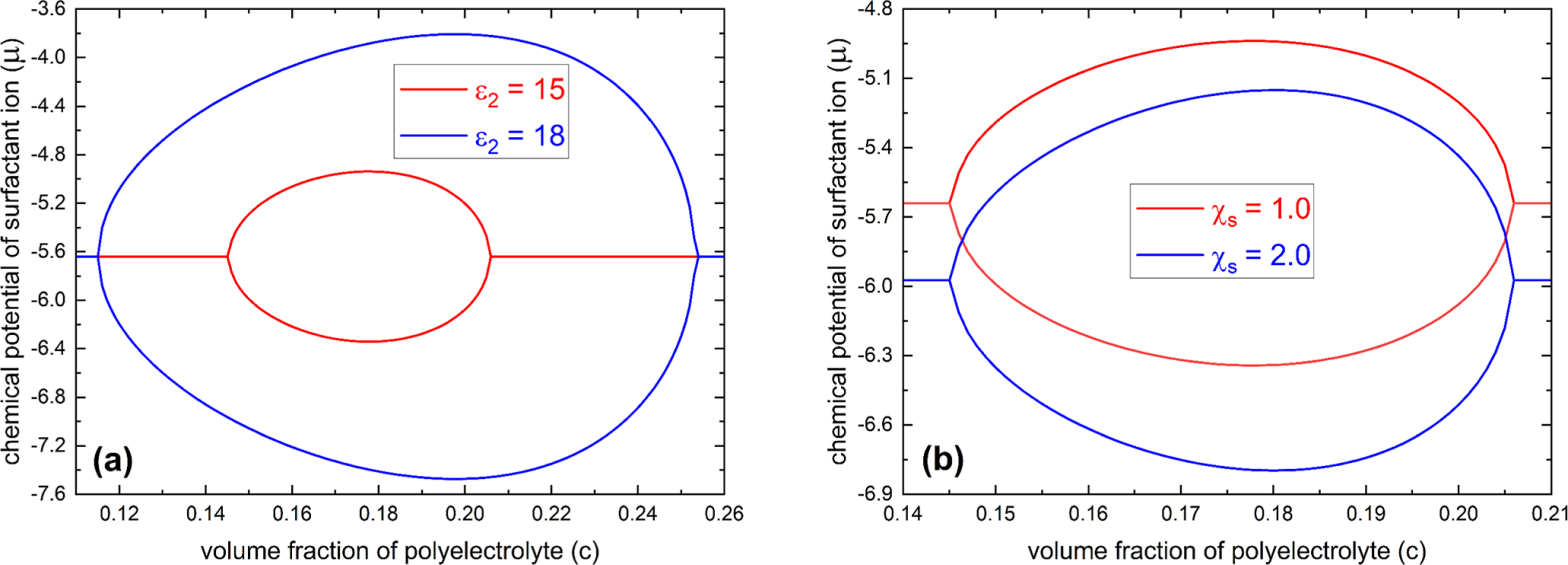
(a) According to eq 16, spinodal phase diagrams of a polyelectrolyte in the dilute solution of an oppositely charged surfactant for different strengths of hydrophobic-aggregation parameter (ε_2_) under the condition of *p* = 0.6, *l*_B_/*a* = 2, ε_1_ = 6, ε_FH,1_ = ε_FH,2_ = 0.4, λ = 1, χ_s_ = 1, and *N* → ∞. (b) According to eq 16, spinodal phase diagrams of a polyelectrolyte in the dilute solution of an oppositely charged surfactant for different demixing effects between the surfactant and water (χ_s_) under the condition of *p* = 0.6, *l*_B_/*a* = 2, ε_1_ = 6, ε_2_ = 15, ε_FH,1_ = ε_FH,2_ = 0.4, λ = 1, and *N* → ∞.

Interestingly, by an increase in charge fraction (*p*) of a polyelectrolyte chain while keeping other model parameters unchanged, the corresponding spinodal phase diagrams (similar to Figure 6a; also see eq 16) indicate that the coexistence region of the collapse transition shifts to a lower concentration of the surfactant, but the coexistence region of the reentry transition shifts to a higher concentration of the surfactant. It is noticeable that this analytical result is corroborated in detail by experimental studies of a recent dissertation^90^ on the phase behaviors of poly­(acrylic acid) and sodium polyacrylate in aqueous solutions of hexadecyl trimethylammonium bromide.

### 3.5 An Estimation of the Critical Aggregation Concentration (CAC)

We note that during the process of charge neutralization between a polyelectrolyte and an oppositely charged surfactant, a strong electrostatic dipole between a monovalent ionic monomer and a monovalent surfactant ion can form because of a noticeable reduction of the dielectric constant around the polyelectrolyte chains, arising from the long alkyl surfactant tails.^21,22^ This indicates a significant shift of ε_1_ on the left-hand side of the spinodal construction, i.e., eq 16, which is often not small for a polyelectrolyte (on the order of about 5 *k*_B_ *T* for the strength of an ionic bond^91^ in water at low salt concentrations and on the order of about 10 *k*_B_ *T* for the strength of the electric dipole interaction^92^). This fact implies that eq 16 melts down when the chemical potential μ is far away from zero, which is approximately given by the condition that the external adsorption field *U* is close to zero, i.e., eq 19. This way, we can estimate the critical aggregation concentration (CAC) of the surfactant in the surfactant–polyelectrolyte binding isotherm for the occurrence of a reentrant condensation, which is given by

20 $$\ln\left(CAC\right)\approx \mu_{A}∼\mu_{C}=-\frac{\varepsilon_{1}}{\lambda }-\frac{\chi_{s}}{3}+\frac{1+\ln2-\ln3}{\lambda }+\ln3-1$$

We can extract some interesting physics from eq 20. First, the larger size of the ionic head of a surfactant ion (λ) can promote the CAC to a higher surfactant concentration. It is remarkable that this analytical prediction was observed in previous experimental studies^54,63,64^ on the reentrant condensation of some polyelectrolytes. Second, if the values of ε_1_ and χ_s_ are sufficiently large, as, for example, with a rather moderate choice of parameters ε_1_ = 6, χ_s_ = 1, and λ ≈ 1, the surfactant concentration which permits a polyelectrolyte reentrant condensation to occur is in the order of about (*n* + 1)*c*_*x*_ ∼ CAC ≈2.0 × 10^–3^ (volume fraction in solution). It is remarkable that this CAC value is lower than the bulk critical micelle concentration (CMC) of some ionic surfactants reported in literature.^93^ We note that this can be the case for both the collapse and reentry branches of the reentrant condensation, as shown from Figures 4–6. According to this simple estimation, we already see that the ionic surfactant concentrations for polyelectrolyte reentrant condensation can be lower than its bulk critical micelle concentration (CMC) even if we do not evaluate the CMC of an ionic surfactant by its molecular structure parameters (*a*, *n*, λ, and ε_2_ in our model; see Section B of Supporting Information for details). Third, because λ ≈ 1 and ε_1_ ≫ χ_s_/3 for most common cases, by eq 20, we see that the primary factor to induce both collapse and reentry transitions of polyelectrolytes at low surfactant concentrations is the strong electrostatic adsorption between ionic monomers and surfactant ions (ε_1_). Notice that these predictions concur with all experimental observations reported in the literature^8–16^ on the reentrant condensation of polyelectrolytes in dilute aqueous solutions of oppositely charged surfactants.

## 4 Conclusions and Remarks

In summary, in this work, we explore the reentrant condensation of polyelectrolytes in the presence of an oppositely charged surfactant, a phenomenon whose phase-transition mechanism is of fundamental importance to the understanding of liquid–liquid phase separation (LLPS) in soft materials and biological systems. Compared with previous theoretical formalisms,^18,28,40–53^ the novelty of the present work is that we focus on the adsorption and attraction effects of surfactants near/on polymer chains and ignore their own nonessential mixing effects if surfactant molecules are far away from polymer chains. This novel approach allows us to construct a simple mean-field equilibrium theory and to solve it analytically with closed-form solutions, which can rationalize the essential features (such as the emergent “egg shape” of spinodal and binodal phase diagrams) of the reentrant condensation of a polyelectrolyte induced by diluted oppositely charged surfactants. This approach also allows us to clearly address the fact that a strong electrostatic adsorption between the ionic monomers and surfactant ions is critical to understand the peculiar phenomenon that both the collapse and reentry transitions of polyelectrolytes can even occur when the concentration of the surfactant is below its bulk critical micelle concentration (CMC). The analytical solution of the theory also indicates that a minimum coupling energy for the nonlinear hydrophobic-aggregation effect of adsorbed surfactants is essential for a phase transition to occur, which explains why polyelectrolytes show that phase transition only if the surfactant chain length is above a certain threshold value.^55–58^

In contrast to uncharged linear polymers,^21,74,75^ a distinctive finding of the present theory in this work is that it is hard to realize a real dilute polymer phase when the fraction of charged monomers is not sufficiently low. This is clear from our theoretical calculations for the limiting case of an infinite chain length. Our theory justifies that the monomer charge is an important factor in the regulation of polymer liquid–liquid phase separation. In addition, we found that it is not necessary to stick to the concept of charge compensation to explain the reentrant condensation of polyelectrolytes, which has been confirmed in detail in previous experimental studies.^66,67^ Our theory showed that it can flexibly realize charge compensation and charge regulation of polyelectrolytes in phase transitions by changing the adsorption strength of surfactant ions on ionic monomers (ε_1_), the demixing strength between the surfactant and water (χ_s_), and the size of the ionic head of a surfactant (λ).

Nevertheless, it is necessary to point out that our approximations neglect contributions that would describe the surfactant’s bulk phase transitions, including micellar formation. The surfactant’s entropic mixing terms are included in the free energy function, but these contributions would hamper the simplicity of the analytical solutions of the model. We have thus, for the sake of clarity and transparency of the formalism, neglected these terms in our analytical approximations and rather focused on systems in which polyelectrolyte condensation occurs at concentrations well below the critical micellar concentration of a surfactant. Another consequence of the restriction to low surfactant concentrations is that the surfactant–polymer interaction cannot be weak, i.e., the adsorption strength of surfactant ions on ionic monomers is not small (ε_1_ ∼ 5 *k*_B_ *T*). For the case of weak interaction (ε_1_ ∼ 1 *k*_B_ *T*), a high ionic surfactant concentration would be required to induce a reentry transition, which can be well-explained by classical polyelectrolyte theories.^21–23^ We also point out that a limitation of the current theory is its neglect of the charge distribution on the polyelectrolyte chains. This approach simplifies our analytical calculations but underestimates the overall entropic effect, which predicts symmetric collapse and reentry transitions, which are, however, not always the experimental observation. A starting point of theoretically accounting for the charge distribution of polyelectrolyte chains can be the calculation framework summarized by Avni, Andelman, and Podgornik.^94^

Another limitation of the current theory is that it has neglected the interaction between the polyelectrolyte backbone and the hydrophobic tail of the surfactant. This implies that our simplified formalism is primarily confined to a hydrophilic polyelectrolyte backbone, i.e., in the case of $\varepsilon_{FH,1}<\frac{1}{2}{\left(1+\frac{1}{\sqrt{N}}\right)}^{2}$ and $\varepsilon_{FH,2}<\frac{1}{2}{\left(1+\frac{1}{\sqrt{N}}\right)}^{2}$ . This approach simplifies our analytical calculations but underestimates the overall hydrophobic effect in the polyelectrolyte phase transition, which may limit its predictions on the phase behaviors of certain amphiphilic polyelectrolytes in the presence of surfactants. For example, the hydrophobic effect can affect and decrease considerably the surfactant concentration that is necessary to trigger the reentry transition, as pointed out by previous experiments.^20,39^ Under certain conditions, the hydrophobic effect may lead to a liquid-like polyelectrolyte phase in coexistence with a solid-like polyelectrolyte phase (liquid–solid phase separation or LSPS; see two classical review papers on this topic^95,96^ ), which is beyond the scope of our simplified theoretical formalism. In principle, for the case of polyelectrolyte backbones with strong hydrophobic properties, our theoretical approach can be refined and extended to accommodate additional hydrophobic adsorption between the surfactant tail and the polymer backbone as well as the related electrostatic repulsion effect among adsorbed surfactant ions. However, a detailed investigation of these above aspects to refine our theory is worthy of future consideration and lies beyond the goal of the present study.

## Supplementary Material



## Notes

Construction of (ε_2_)_min_ with a higher numerical precision and estimation of the bulk critical micelle concentration (CMC) of an ionic surfactant (PDF)
