# Supplementary material for: An Analytical Understanding of Reentrant Condensation of a Polyelectrolyte in the Presence of an Oppositely Charged Surfactant
Source: Langmuir. 2025 Jul 22;41(30):19683–97. doi: 10.1021/acs.langmuir.5c01217 (PMC12333356; doi:10.1021/acs.langmuir.5c01217)
Supplement: Supplementary file 1 [file la5c01217_si_001.pdf]

Supporting Information

An analytical understanding of reentrant condensation of a polyelectrolyte in the presence of an oppositely-charged surfactant

Huaisong Yong[1,2,3*], Holger Merlitz[3]

[1]School of New Energy and Materials, Southwest Petroleum University, 610500 Chengdu, China

[2]Department of Molecules & Materials, MESA+ Institute, University of Twente, 7500 AE Enschede, The Netherlands

[3]Institute Theory of Polymers, Leibniz-Institut für Polymerforschung Dresden e.V., D-01069 Dresden, Germany

[*]Correspondence author: Huaisong Yong (yonghs@swpu.edu.cn; h.yong@utwente.nl; yonghuaisong@gmail.com)

Number of pages: 7

Number of figures: 2

Number of schemes: 0

Number of tables: 4

Table of contents

Section A: A construction of $(\varepsilon_2)_{min}$ with a higher numerical precision 2

Section B: An estimation of bulk critical micelle concentration (CMC) of an ionic surfactant........ 7

Supporting references 7

Section A: A construction of $(\varepsilon_2)_{\min}$ with a higher numerical precision

The primary goal of this section is to get a construction of $(\varepsilon_2)_{\min}$ with a higher numerical precision.

Here, we quote **Equation(4)** of the main text for convenience,

$$0=\frac{d(2\chi_0)}{dc}=-\frac{1}{Nc^2}-\frac{p}{c^2}+\frac{(1+p)^3}{\left[1-(1+p)c\right]^2}+\frac{3\sqrt{\pi}}{8}\left(\frac{l_B}{a}\frac{p}{c}\right)^{\frac{3}{2}} \quad \text{(S1)}$$

After some basic algebraic manipulations, we get the following equation,

$$0=f(c)\equiv-\left(\frac{1}{N}+p\right)\left[1-(1+p)c\right]^2+(1+p)^3c^2+\frac{3\sqrt{\pi}}{8}\left(\frac{l_B}{a}p\right)^{\frac{3}{2}}\left[1-(1+p)c\right]^2\sqrt{c} \quad \text{(S2)}$$

Equation(S2) is a quintic equation of $\sqrt{c}$, which does not exist an exact explicit analytical solution for c according to the well-known *Abel–Ruffini theorem*. Here we define the function $f(c)$ for a convenience of graphical presentation in **Figure S1**. We note that there is a physical constraint on the fraction of charged monomers (p) in our model, i.e, $0 \le p \le 1$. This property indicates that we can get the following limiting solution for **Equation(S2)** for a very small value of p by ignoring the third term, since the third term is much smaller than the sum of other terms for this case.

$$\frac{1}{c_0}=(1+p)+\frac{(1+p)^{\frac{3}{2}}}{\left(\frac{1}{N}+p\right)^{\frac{1}{2}}}>1 \quad \text{with} \quad c_0=\frac{\left(\frac{1}{N}+p\right)+\sqrt{\left(\frac{1}{N}+p\right)(1+p)}}{(1+p)\left(\sqrt{\frac{1}{N}+p}+\sqrt{1+p}\right)^2}<1 \quad \text{(S3)}$$

Note that the exact solution of **Equation(S2)** is recovered by **Equation(S3)** when the parameter p is close to zero. However, as shown in **Figure S1** and **Tables S1-S4**, there is a large deviation in **Equation(S3)** from the numerical solution of c if p is obviously larger than zero and the polymer chain is very long ($N \to \infty$). This shortcoming can be overcome by using the method of fixed-point iteration [1] with the initial value defined by **Equation(S3)**.

In order to employ the method of fixed-point iteration [1] to obtain a good approximation solution of c for the limiting case of $p \to 1$, we re-organize **Equation(S2)** into the following ***master equation for the fixed-point iteration***,

$$2\left(\frac{1}{N}+p\right)(1+p)c_{i+1}=\left(\frac{1}{N}+p\right)+\left(\frac{1}{N}-1\right)(1+p)^2c_i^2-\frac{3\sqrt{\pi}}{8}\left(\frac{l_B}{a}p\right)^{\frac{3}{2}}\left[1-(1+p)c_i\right]^2\sqrt{c_i} \quad \text{(S4)}$$

Here c_{i+1} and c_i are the i+1-th and i-th generation approximations of c respectively. The variable c ($0 < c < 1$) has a higher power exponent on the right-hand side of **Equation(S4)**, this implies that the equation is convergent with the iteration of the variable c which is usually a requirement of the method of fixed-point iteration [1]. By insertion of **Equation(S3)** into the right-hand side of **Equation(S4)**, we get the first-generation estimation of c as

$$c_1 = \frac{\left(\frac{1}{N}+p\right)+\sqrt{\left(\frac{1}{N}+p\right)(1+p)}}{(1+p)\left(\sqrt{\frac{1}{N}+p}+\sqrt{1+p}\right)^2} - \frac{\frac{3\sqrt{\pi}}{16}\left(\frac{l_B}{a}p\right)^{\frac{3}{2}}}{\sqrt{\left(\frac{1}{N}+p\right)(1+p)}\left(\sqrt{\frac{1}{N}+p}+\sqrt{1+p}\right)^{\frac{5}{2}}}$$

$$= c_0 - \frac{\frac{3\sqrt{\pi}}{16}\left(\frac{l_B}{a}p\right)^{\frac{3}{2}}}{\sqrt{\left(\frac{1}{N}+p\right)(1+p)}\left(\sqrt{\frac{1}{N}+p}+\sqrt{1+p}\right)^{\frac{5}{2}}} \quad \text{(S5)}$$

As shown in **Figure S1** and **Tables S1-S4**, we note that the estimation by **Equation(S5)** is significantly improved even for the large cases of $p \to 1$ with $l_B/a = 2.0$ and $N \to \infty$. In general, one can get a higher-and-higher-precision estimation for c by routinely employing the master equation of **Equation(S4)**. However, we will not pursue this goal in this research and we are much interested in the difference for the estimations of $(\varepsilon_2)_{\min}$ by using **Equation(S3)** and **Equation(S5)**. As shown by **Table S3** and **Table S4**, the relative estimation error of **Equation(S5)** is less than 5% and already acceptable for qualitative applications within experimental values of the parameter l_B/a (which is usually on the order of unit).

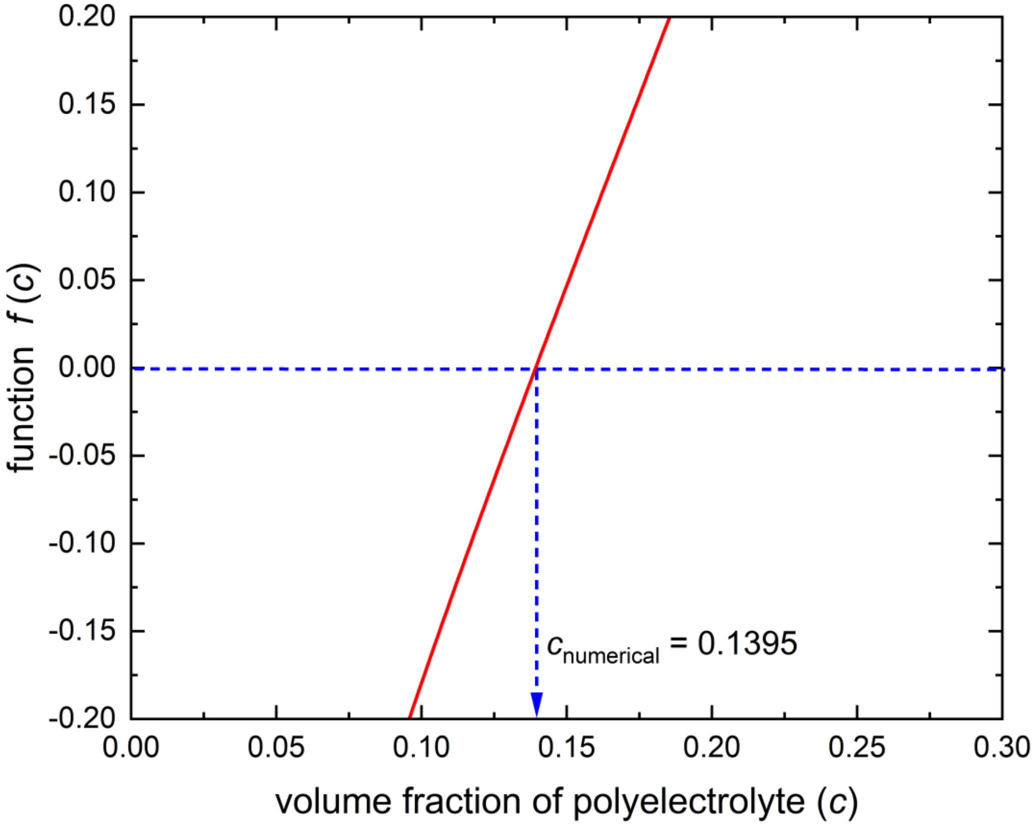

Figure S1. The numerical solution of **Equation (S2)** is $c = 0.1395$ for the parameters $p = 1$, $l_B/a = 2$ and $N \to \infty$. The

analytical estimation by **Equation(S3)** is c = 0.2071 and the analytical estimation by **Equation(S5)** is c = 0.1337. We note that the analytical estimation by the first-generation fixed point internation is already very close to the numerical solution.

Table S1. The solutions of **Equation(S2)** according to **Equation(S3)**, **Equation(S5),** and numerical calculations for the parameters l_B/a = 2 and $N \to \infty$.

	p = 0	p = 0.05	p = 0.1	p = 0.2	p = 0.3	p = 0.4	p = 0.5	p = 0.6	p = 0.7	p = 0.8	p = 0.9	p = 1.0
c_0	0	0.1706	0.2106	0.2416	0.2496	0.2488	0.2440	0.2374	0.2299	0.2222	0.2146	0.2071
c_1	0	0.1443	0.1694	0.1835	0.1828	0.1773	0.1700	0.1623	0.1546	0.1473	0.1403	0.1337
$c_{\mathrm{numerical}}$	0	0.1585	0.1865	0.2026	0.2003	0.1932	0.1834	0.1731	0.1641	0.1554	0.1471	0.1395

Table S2. The solutions of **Equation(S2)** according to **Equation(S3)**, **Equation(S5),** and numerical calculations for the parameters l_B/a = 2 and N = 1.

	p = 0	p = 0.05	p = 0.1	p = 0.2	p = 0.3	p = 0.4	p = 0.5	p = 0.6	p = 0.7	p = 0.8	p = 0.9	p = 1.0
c_0	0.5000	0.4762	0.4545	0.4167	0.3846	0.3571	0.3333	0.3125	0.2941	0.2778	0.2632	0.2500
c_1	0.5000	0.4745	0.4503	0.4068	0.3695	0.3374	0.3097	0.2857	0.2646	0.2461	0.2297	0.2151
$c_{\mathrm{numerical}}$	0.5000	0.4761	0.4512	0.4079	0.3712	0.3383	0.3108	0.2873	0.2636	0.2464	0.2300	0.2150

Table S3. The solutions of **Equation(S2)** according to **Equation(S3)**, **Equation(S5),** and numerical calculations for the parameters l_B/a = 1 and $N \to \infty$.

	p = 0	p = 0.05	p = 0.1	p = 0.2	p = 0.3	p = 0.4	p = 0.5	p = 0.6	p = 0.7	p = 0.8	p = 0.9	p = 1.0
c_0	0	0.1706	0.2106	0.2416	0.2496	0.2488	0.2440	0.2374	0.2299	0.2222	0.2146	0.2071
c_1	0	0.1613	0.1960	0.2211	0.2260	0.2235	0.2179	0.2108	0.2033	0.1957	0.1883	0.1812
$c_{\mathrm{numerical}}$	0	0.1668	0.2022	0.2290	0.2344	0.2321	0.2250	0.2179	0.2106	0.2022	0.1951	0.1873

Table S4. The solutions of **Equation(S2)** according to **Equation(S3)**, **Equation(S5),** and numerical calculations for the parameters l_B/a = 1 and N = 1.

	p = 0	p = 0.05	p = 0.1	p = 0.2	p = 0.3	p = 0.4	p = 0.5	p = 0.6	p = 0.7	p = 0.8	p = 0.9	p = 1.0
c_0	0.5000	0.4762	0.4545	0.4167	0.3846	0.3571	0.3333	0.3125	0.2941	0.2778	0.2632	0.2500
c_1	0.5000	0.4756	0.4530	0.4132	0.3793	0.3502	0.3250	0.3030	0.2837	0.2666	0.2513	0.2376
$c_{\mathrm{numerical}}$	0.5000	0.4761	0.4532	0.4131	0.3800	0.3502	0.3258	0.3037	0.2848	0.2682	0.2533	0.2393

By insertion of **Equation(S5)** into **Equation(3)** of the main text, we get an estimation of the critical or minimum value of χ_0,

$$2\chi_{0,\min} \equiv \frac{8}{27}\varepsilon_2 p^2 + 2\varepsilon_{FH,1}\left(1+p\right)p + 2\varepsilon_{FH,2}\left(1-p^2\right)$$
$$= \frac{1}{Nc} + \frac{p}{c} + \frac{\left(1+p\right)^2}{1-\left(1+p\right)c} - \frac{3\sqrt{\pi}}{4}\left(\frac{l_B}{a}p\right)^{\frac{3}{2}}\frac{1}{\sqrt{c}} \tag{S6}$$

Then we get the minimum of ε_2 by **Equation(S6)** as

$$\left(\varepsilon_2\right)_{\min} \simeq -\frac{27\left[\varepsilon_{FH,1}\left(1+p\right)p + \varepsilon_{FH,2}\left(1-p^2\right)\right]}{4p^2}$$
$$+\frac{27}{8p^2}\left[\frac{1}{Nc} + \frac{p}{c} + \frac{\left(1+p\right)^2}{1-\left(1+p\right)c} - \frac{3\sqrt{\pi}}{4}\left(\frac{l_B}{a}p\right)^{\frac{3}{2}}\frac{1}{\sqrt{c}}\right] \tag{S7}$$

In both **Equation(S6)** and **Equation(S7)** we ignored the subscripts of the variable c to highlight that the structures of these two equations are independent of any iteration procedure. An important feature of the construction of **Equation(S7)** is that: As shown in **Figure S2**, no matter the values of $l_B/a \geq 0$ and $\varepsilon_{FH,1} \geq 0$, there is a unique local maximum of $(\varepsilon_2)_{\min}$ when $\varepsilon_{FH,2}$ is larger than $\frac{1}{2}\left(1+\frac{1}{\sqrt{N}}\right)^2$, but no local maximum of $(\varepsilon_2)_{\min}$ exists when $0 \leq \varepsilon_{FH,2} \leq \frac{1}{2}\left(1+\frac{1}{\sqrt{N}}\right)^2$. In addition, this feature is independent of the estimation accuracy for the solution of c in **Equation(S2)**. From **Figure S2**, we also note that the value of $(\varepsilon_2)_{\min}$ according to **Equation(S3)** is overestimated for large values of p; however, this overestimation disappears when the parameter l_B/a is small (the upper bound is about $l_B/a = 1$).

Based on our above calculations, we conclude that the simple approximation solution by **Equation(5)** in the main text, i.e., also **Equation(S3)** in this appendix, is sufficient to deduce the key features of the overall effective interaction parameter χ_0 and $(\varepsilon_2)_{\min}$ without compromising on the physical conclusions. Thus, to get a simple enough expression for $(\varepsilon_2)_{\min}$ in the main text, we do not use the higher precision solution of c such as by **Equation(S5)**.

As close of this section, we would like to point out that one can always improve the accuracy of $(\varepsilon_2)_{\min}$ as one like by employing the method of fixed-point iteration reported in this section. However, with a higher-and-higher-precision estimation for c, the explicit analytical expression for $(\varepsilon_2)_{\min}$ will become cumbersome and hard to handle, which is a typical technical problem in the application of the method of fixed-point iteration [2-3]. This technical problem can be overcome by further using the techniques of symbolic regression [4], but a detailed investigation of this aspect is obviously beyond the goal of the present study.

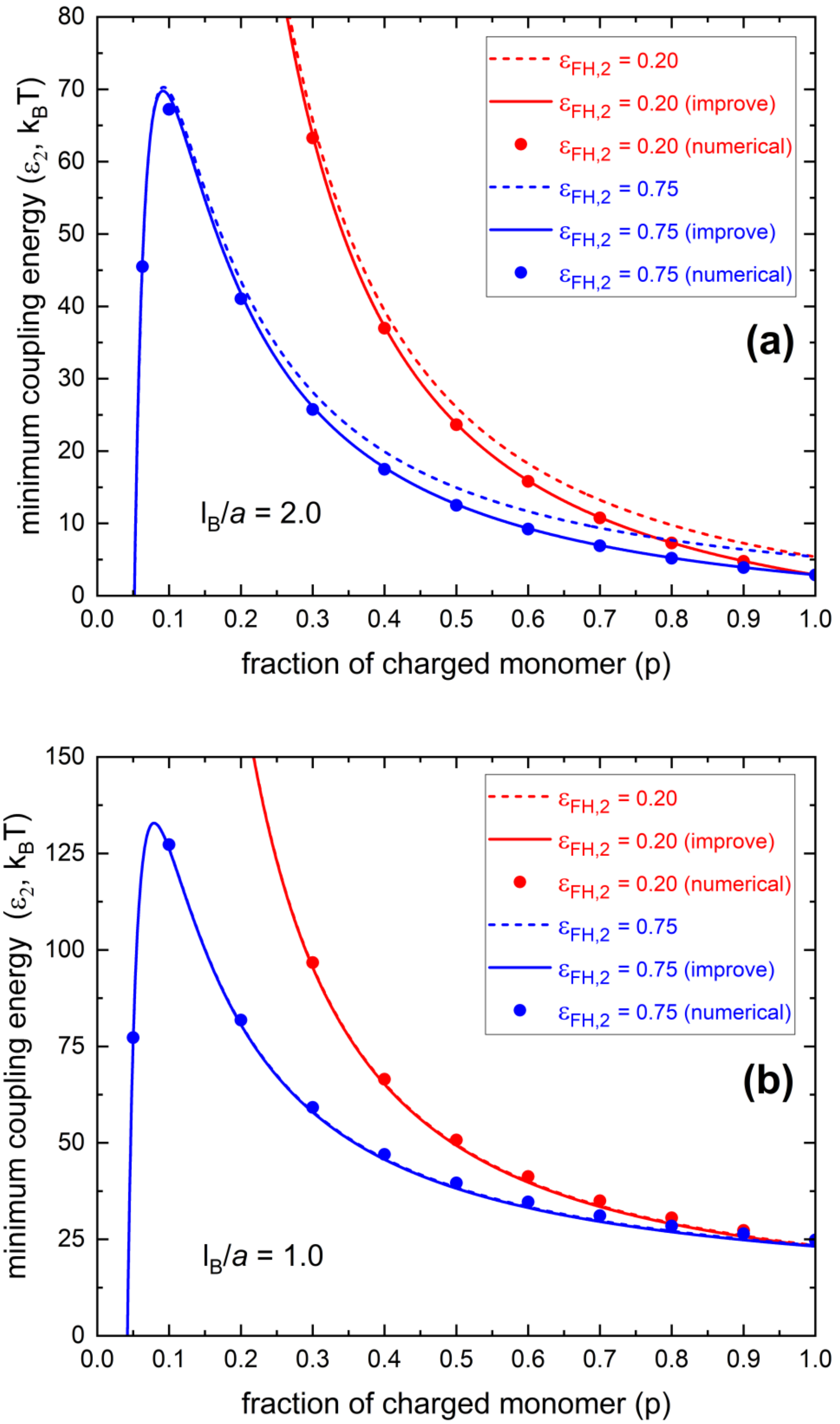

Figure S2. According to **Equation(S7)**, the minimum coupling energy (ε_2) with respect to the fraction of charged monomer (p) for typical values of the parameter $\varepsilon_{\mathrm{FH},2}$ with $\varepsilon_{\mathrm{FH},1} = 0.45$ and $N \to \infty$, where $l_B/a = 2$ for panel **a** and $l_B/a = 1$ for panel **b**. The dashed lines in the figure are calculations according to **Equation(S3),** the solid lines are calculations according to **Equation(S5)**, and the filled circles are calculations according to the numerical solutions of **Equation(S2)**.

Section B: An estimation of bulk critical micelle concentration (CMC) of an ionic surfactant

The bulk critical micelle concentration (CMC) of an ionic surfactant in our model can be roughly evaluated on the order of about

$$\ln\left(CMC\right) \sim -\frac{\varepsilon_2}{2}. \quad \text{(S8)}$$

See chapter eight of the monograph [5] by de Gennes, Brochard-Wyart and Quéré for details of related evaluation methods. Its more accurate value can be considered in detail according to molecular structure parameters (a, n, λ, and ε_2 in our model) by advanced methods reported in literature, such as the graph convolutional neural networks [6] and the self-consistent field theory [7]. To simplify our discussion, we do not consider it in detail in this research.

Supporting references

1. Granas, A.; Dugundji, J., *Fixed Point Theory*. Springer: New York, USA, 2003.
2. Qian, D.; Michaels, T. C. T.; Knowles, T. P. J., Analytical Solution to the Flory–Huggins Model. *J. Phys. Chem. Lett.* **2022,** *13* (33), 7853-7860.
3. Souza, J. P. d.; Stone, H. A., Exact analytical solution of the Flory–Huggins model and extensions to multicomponent systems. *J. Chem. Phys.* **2024,** *161*, 044902
4. Affenzeller, M.; Burlacu, B.; Kommenda, M.; Kronberger, G.; Winkler, S. M., *Symbolic Regression*. CRC Press: Milton Park, Abingdon, Oxon, UK, 2024.
5. de Gennes, P.-G.; Brochard-Wyart, F.; Quéré, D., *Capillarity and Wetting Phenomena: Drops, Bubbles, Pearls, Waves*. Springer: New York, USA, 2004.
6. Qin, S.; Jin, T.; Van Lehn, R. C.; Zavala, V. M., Predicting Critical Micelle Concentrations for Surfactants Using Graph Convolutional Neural Networks. *J. Phys. Chem. B* **2021,** *125* (37), 10610-10620.
7. Duan, C.; Wang, M.; Ghobadi, A.; Eike, D. M.; Wang, R., Quantifying the Critical Micelle Concentration of Nonionic and Ionic Surfactants by Self-Consistent Field Theory. *arXiv preprints* **2024**.
